# Fibronectin fragments generated by pancreatic trypsin act as endogenous inhibitors of pancreatic tumor growth

**DOI:** 10.1186/s13046-023-02778-y

**Published:** 2023-08-09

**Authors:** Andrea Resovi, Perla Persichitti, Laura Brunelli, Lucia Minoli, Patrizia Borsotti, Giulia Garattini, Matteo Tironi, Erica Dugnani, Miriam Redegalli, Giulia De Simone, Roberta Pastorelli, Maria Rosa Bani, Lorenzo Piemonti, Deane F. Mosher, Raffaella Giavazzi, Giulia Taraboletti, Dorina Belotti

**Affiliations:** 1 Department of Oncology, Istituto di Ricerche Farmacologiche Mario Negri IRCCS, Bergamo and Milan, Italy; 2 Department of Environmental Science, Istituto di Ricerche Farmacologiche Mario Negri IRCCS, Milan, Italy; 3 Department of Biomedical Engineering, Istituto di Ricerche Farmacologiche Mario Negri IRCCS, Bergamo, Italy; 4 Diabetes Research Institute, IRCCS Ospedale San Raffaele, Milano, Italy; 5 Departments of Biomolecular Chemistry and Medicine, University of Wisconsin, Madison, WI USA

**Keywords:** PDAC, Fibronectin, Trypsin, FAK, FGFR

## Abstract

**Background:**

The pancreatic microenvironment has a defensive role against cancer but it can acquire tumor-promoting properties triggered by multiple mechanisms including alterations in the equilibrium between proteases and their inhibitors. The identification of proteolytic events, targets and pathways would set the basis for the design of new therapeutic approaches.

**Methods and results:**

Here we demonstrate that spheroids isolated from human and murine healthy pancreas and co-transplanted orthotopically with pancreatic ductal adenocarcinoma (PDAC) in mouse pancreas inhibited tumor growth. The effect was mediated by trypsin-generated fibronectin (FN) fragments released by pancreatic spheroids. Tumor inhibition was observed also in a model of acute pancreatitis associated with trypsin activation. Mass spectrometry proteomic analysis of fragments and mAb against different FN epitopes identified the FN type III domain as responsible for the activity. By inhibiting integrin α5β1, FAK and FGFR1 signaling, the fragments induced tumor cell detachment and reduced cell proliferation. Consistent with the mutual relationship between the two pathways, FGF2 restored both FGFR1 and FAK signaling and promoted PDAC cell adhesion and proliferation. FAK and FGFR inhibitors additively inhibited PDAC growth in vitro and in orthotopic in vivo models.

**Conclusions:**

This study identifies a novel role for pancreatic trypsin and fibronectin cleavage as a mechanism of protection against cancer by the pancreatic microenvironment. The finding of a FAK-FGFR cross-talk in PDAC support the combination of FAK and FGFR inhibitors for PDAC treatment to emulate the protective effect of the normal pancreas against cancer.

**Supplementary Information:**

The online version contains supplementary material available at 10.1186/s13046-023-02778-y.

## Background

The normal tissue microenvironment plays a major role in the maintenance of organ structure, tissue specificity, and homeostasis, and in suppressing malignant progression [1]. Parenchymal cells, fibroblasts, endothelial and inflammatory cells all communicate with each other through secreted extracellular matrix molecules and growth factors [2]. Negative feedback loops maintain the correct balance between the different cell types and avoid uncontrolled cell proliferation. In certain circumstances, particularly following infection, inflammation, trauma or other insults, the microenvironment undergoes profound alterations which can reestablish homeostasis or, conversely, promote disease progression [3, 4].

Pancreatic ductal adenocarcinoma (PDAC) is one of the most lethal tumors in the western world. It involves deregulated extracellular matrix deposition and abundant fibrosis, with qualitative and quantitative alterations in the equilibrium between proteases and inhibitors. Extracellular proteases originally considered favorable to malignancy, have a much more complex role than previously assumed, with some enzymes acting in an opposing fashion to block cancer growth and maintain tissue homeostasis [5].

Trypsinogen is zymogen of one of the major pancreatic enzymes secreted by exocrine acinar cells. In physiological conditions, its conversion to trypsin by enterokinase and the digestive activity of trypsin are closely regulated to prevent tissue damage [6]. Events causing acinar cell damage lead to premature enzyme activation and secretion [6]. Depending on the context, trypsin can have pro- or anti- tumor effects by directly triggering specific cell signaling pathways or by generating biologically active fragments of extracellular matrix proteins that cooperate with – or antagonize – the effect of growth factors in tumor progression [7, 8].

Here, to investigate the role of the pancreatic microenvironment in PDAC progression, we studied the morphological and functional changes in PDAC cells induced by spheroids isolated from human and murine healthy pancreas. We found that the pancreatic microenvironment counteracted the growth of PDAC through the activity of trypsin-generated FN fragments, pointing to a role of proteases and protease-generated extracellular matrix fragments in the control of tumor growth. Finally, the involvement of the FGF2/FGFR1 and integrin β1/FAK pathways in this process supports the use of combinations of FAK and FGFR inhibitors in PDAC treatment.

## Methods

### PDAC models

#### Tumor cells

The MIAPaCa2 and BxPC3 human pancreatic cancer cell lines were obtained, respectively, from Istituto Zooprofilattico Sperimentale (Brescia, Italy) and from American Type Culture Collection (ATCC) and authenticated using the AmpFlSTR® Identifiler® PCR Amplification Kit (Applied Biosystems, Merk, Milano, Italy). The FC1199 pancreatic cancer cell line [9] was kindly provided by D.A. Tuveson (Cold Spring Harbor, NY, USA). MIAPaCa2, FC1199 and BxPC3 were cultured respectively in Dulbecco modified Eagle medium (DMEM) and RPMI 1640 Medium (ATCC modification) (Gibco, ThermoFisher, Rodano, Italy) supplemented with 10% FCS (Euroclone, Milano, Italy) and 1% L-glutamine (Gibco) and were routinely tested for mycoplasma infection. Stocks of cell lines were stored frozen in liquid nitrogen and kept in culture for no more than 3 weeks.

#### Pancreatic spheroids

Human spheroids were obtained from the exocrine pancreas of human donors after pancreatic islet isolation [10] in the Pancreatic Islet Processing Unit, San Raffaele Scientific Institute, Milan, Italy. Their collection and use was approved by the local scientific ethic committees. Murine spheroids were obtained as follows: pancreatic fragments from four-months-old female C57BL/6 mice (Charles River Laboratories, Lecco, Italy) were soaked for ten minutes in cold P-collagenase (1 mg/mL, Roche, Monza, Italy) and then incubated at 37 °C for 15 min under agitation. After two washes with cold Hank’s Balanced Salt Solution (HBSS) (Gibco), cells were filtered with a 500 μm strainer and washed again. After removing islets by centrifugation density gradient, isolated cells were left to spontaneously aggregate and form spheroids in non-adherent tissue culture plates. Cell vitality was checked using Trypan Blue (Sigma-Aldrich, Merk, Milano, Italy).

#### Preparation of spheroid conditioned media

Spheroids were seeded in serum-free DMEM with or without trypsin inhibitors: aprotinin (1.7 µg/mL, Sigma-Aldrich) or Tosyl-L-lysyl-chloromethane hydrochloride (TLCK 50–100 µM, abcam, Cambridge, UK). After 24 h spheroids were washed in serum-free DMEM and reseeded with or without inhibitors. After 72 h, conditioned media were collected, centrifuged at 3000 rpm for 10 min at 4 °C, and stored frozen at -80 °C.

#### In vivo tumor models

Mice were maintained under specific pathogen-free conditions and handled using aseptic procedures. Procedures involving animals and their care were conducted in conformity with institutional guidelines that comply with national (Lgs 26/2014) and EU directives laws and policies (EEC Council Directive 2010/63, in line with guidelines for the welfare and use of animals in cancer research [11]. Animal studies were approved by the Mario Negri Institute Animal Care and Use Committee and by the Italian Ministry of Health (DM 85/2013-B and Authorization no.519/2021-PR and no.125/2016-PR).

Patient-derived PDAC xenograft HuPa4 (5 × 10^5^ cells), MIAPaCa2-luc and FC1199 (5 × 10^4^ cells) were injected with or without a suspension of spheroids (at the estimated ratio of 1:300) in the pancreas of six- to eight-week-old female severe combined immunodeficiency (SCID) mice for HuPA4 [12] (Envigo, Correzzana, Italy), or female athymic Foxn1 nu/nu mice (Envigo) MIAPaCa2-luc, or female C57BL/6 mice for FC1199.

The experiments were concluded when the first animals showed sign of suffering. After euthanasia the pancreas was weighted and collected for further analysis.

To test the effects of spheroid conditioned media, HuPa4 cells (2 × 10^6^) were injected subcutaneously in the flank of six- to eight-week-old male severe combined immunodeficiency (SCID) mice. Mice were injected subcutaneously with spheroid conditioned or control medium (200 µL) 24 h before tumor injection, and five days a week until euthanasia. Subcutaneous tumor growth was monitored three times a week with a digital caliper, and tumor volume (mm^3^) was calculated as [length (mm) x width^2^ (mm^2^)/2].

### De-adhesion and proliferation assay

MIAPaCa2 and FC1199 cells were seeded in 96-well plates (3000/well) in complete medium. After 24 h, different stimuli were added as indicated and incubated for 1 h (de-adhesion) or 72 h (proliferation). De-adhesion was measured with crystal violet (Sigma-Aldrich). Proliferation was measured with MTS (Promega, Madison, Wisconsin, USA) or crystal violet. Each condition was tested in triplicate. Data are expressed as percentages of control (absence of stimuli).

### Immunofluorescence

MIAPaCa2 cells were seeded in 8-well ibi-treat micro-slides (Ibidi, Giemme, Milano, Italy) (30,000/well) in DMEM with 10% FCS. After 48 h, different stimuli were added and incubated for the times indicated. Cells were then fixed with 2% PFA (Sigma-Aldrich) in 4% sucrose for 10 min at room temperature (RT), permeabilized with 0.1% Triton X-100 (Biorad, Segrate, Italy) for 3 min and then incubated for 30 min with 3% BSA (Sigma-Aldrich) at RT. The samples were stained with the primary antibody diluted in 3% BSA overnight at 4 °C followed by incubation with the appropriate secondary antibody for 1 h at RT. Actin was stained with rhodamine phalloidin (Invitrogen, ThermoFisher) at the dilution of 1:40 for 1 h at RT. Nuclei were counterstained with DAPI for 5 min at RT. Images were taken using a Leica SP8 confocal microscope (Leica Microsystems, Wetzlar, Germany).

### Trypsin digestion of fibronectin

50 µg of human plasma fibronectin (FN, Sigma-Aldrich) was digested at 1:50 (w/w) with trypsin (Trypsin Sequencing Grade, Sigma-Aldrich) for 10, 60 and 240 min at 37 °C with gentle agitation. Proteolysis was stopped by the addition of TLCK 800 µM (abcam) at 4 °C.

### Western blot

Cells were lysed with RIPA buffer (ThermoFisher), containing protease inhibitors (Roche) and phosphatase inhibitors (Sigma-Aldrich) and centrifuged at 12000xg at 4 °C.

Proteins were separated by 4–12% SDS-PAGE (Genscript, Twin Helix, Rho, Italy) under reducing conditions and transferred to nitrocellulose membranes (GE Healthcare, Milano, Italy). After blocking with 5% BSA in Tris-buffered saline (TBS) 0.1% Tween-20, membranes were incubated with primary antibodies in 2% BSA overnight at 4 °C, followed by IR- or peroxidase-labeled secondary antibody. Alpha-tubulin 1:2000 or GAPDH 1:2500 (Sigma-Aldrich) antibodies were used to confirm equal loading. Signals were detected using an Odyssey FC Imaging System (LI-COR,Biosciences, Lincoln, Nevada, USA). Bands were quantified using Image Studio Lite 5.0 (LI-COR) software.

### Proteomics

Label-free proteomics of inhibitory and stimulatory CM was carried out as previously described [13] and detailed in the supplementary material.

### Statistical analysis

Statistical analysis was done with GraphPad Prism 9.3.1 (GraphPad, La Jolla, CA). Differences in tumor growth were analyzed by two-way ANOVA followed by Bonferroni’s multiple comparison test. For other analysis, a nonparametric Mann-Whitney test was used for comparison of two groups, and one-way ANOVA, followed by Dunnett’s or Tukey’s multiple comparison test, for three or more groups. P-values less than 0.05 were considered statistically significant.

## Results

### Spheroids from normal human pancreas inhibit PDAC cell growth in vivo

**Fig. 1:**
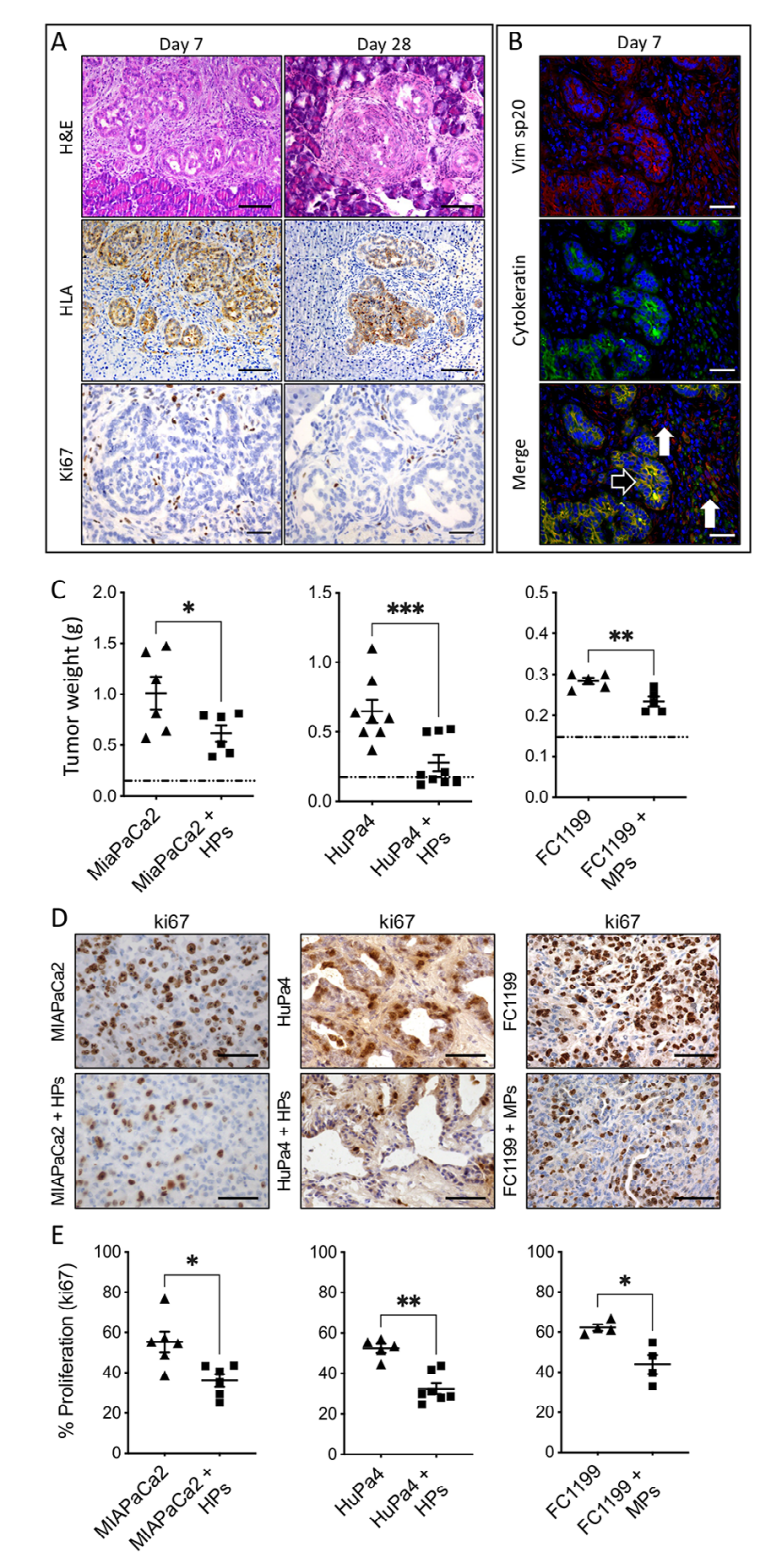
Pancreatic spheroids inhibit orthotopic tumor growth of co-transplanted PDAC tumors. **(A)** H&E staining, HLA (200X, scale bar 100 μm) and Ki67 (400x, scale bar 50 μm) immuno-staining of human pancreatic spheroids (HPs) transplanted in the pancreas of immunodeficient mice at 7 and 28 days after HPs injection. **(B)** Double immunofluorescence of anti-human vimentin (mesenchymal cells) and anti-cytokeratin (epithelial cells). Black arrow indicates human epithelial cells co-expressing cytokeratin and vimentin 7 days after HPs injection. White arrows indicate human vimentin-expressing fibroblasts (400x, scale bar 100 μm). **(C)** Human (HPs) and murine (MPs) pancreatic spheroids inhibit orthotopic tumor growth of co-transplanted PDAC tumors. Tumor weight at euthanasia (MIAPaCa2 61 days post-injection, HuPa4 136 days post-injection, FC1199 14 days post-injection). The mean weight of a healthy pancreas is indicated by the dotted line (mean ± SEM; 5 for each group, one-way ANOVA with Tukey’s multiple comparison test, *p < 0.05,**p < 0.005, ***p < 0.0005). **(D-E)** Ki67 immunostaining and quantification of tumor cell proliferation at euthanasia (400X, scale bar 50 μm) (mean ± SEM; 4 for each group, Mann-Whitney, *p < 0.05,**p < 0.005)

Tumor cells and their neighboring non-tumor cells establish complex reciprocal interactions that have a variety of effects on tumor progression [14]. To investigate the role of the pancreatic microenvironment on PDAC progression, we isolated the exocrine portion of normal human pancreas from healthy donors after separation of pancreatic islets. The resulting cell preparations were composed of acinar cells (ESA^+^, CA19.9^−^), ductal cells (ESA^+^, CA19.9^+^), and mesenchymal and inflammatory cells, (ESA^−^ and CA19.9^−^) (Supplementary Fig. 1 shows four representative clusters). Both acinar and ductal cells express the epithelial marker ESA (www.proteinatlas.org/ENSG00000119888-EPCAM/tissue/pancreas) but only ductal cells express also CA19.9, analyzed by FACS [15]. Cells cultured over five days under non-adhesive conditions spontaneously formed 3D spheroids mimicking the cell interactions of the original pancreas [10]. To assess the suitability of spheroids for in vivo studies we first transplanted them orthotopically in the pancreas of immunodeficient mice. Well defined aggregates of human (HLA+) cells were detected 7 and 28 days after injection; their proliferation activity was low but persistent in time (from 0 to 4 ki67-positive nuclei per single HPF) indicating that spheroids survive for at least 4 weeks after the injection (Fig. 1A). Small aggregates of HLA positive cells were observed up to 90 days post-injection (data not shown). In terms of the cell composition, human specific anti-cytokeratin and anti-vimentin antibodies identified both epithelial cells and fibroblasts in the surviving clusters (Fig. 1B).

To assess their influence on tumor growth, spheroids from human pancreas (HPs) were co-transplanted orthotopically in the pancreas of immunodeficient mice with the human MIAPaCa2 PDAC cells. After two months, the tumors co-transplanted with HPs were significantly smaller than the ones transplanted without HPs (p < 0.01) (Fig. 1C). HPs had an inhibitory effect on tumor growth also on HuPa4, a patient-derived PDAC xenograft (HuPa4 PDX) (p < 0.0005) [12].

Similar effects were observed in a syngeneic immunocompetent system, using spheroids isolated from mouse normal pancreas (MPs) co-injected with FC1199, a murine PDAC cell line derived from tumors arisen in Kras-P53-Pdx-1-Cre GEM mice (p < 0.005) [9]; this indicates a common molecular mechanism conserved between species at the basis of the inhibition of PDAC growth by normal pancreatic cells (Fig. 1C).

In agreement with the smaller tumor masses, Ki67 immunostaining indicated fewer proliferating cells in tumors co-transplanted with spheroids than in control ones (Fig. 1D and E).

### Inhibition of tumor growth is mediated by soluble molecules released by pancreatic spheroids

**Fig. 2:**
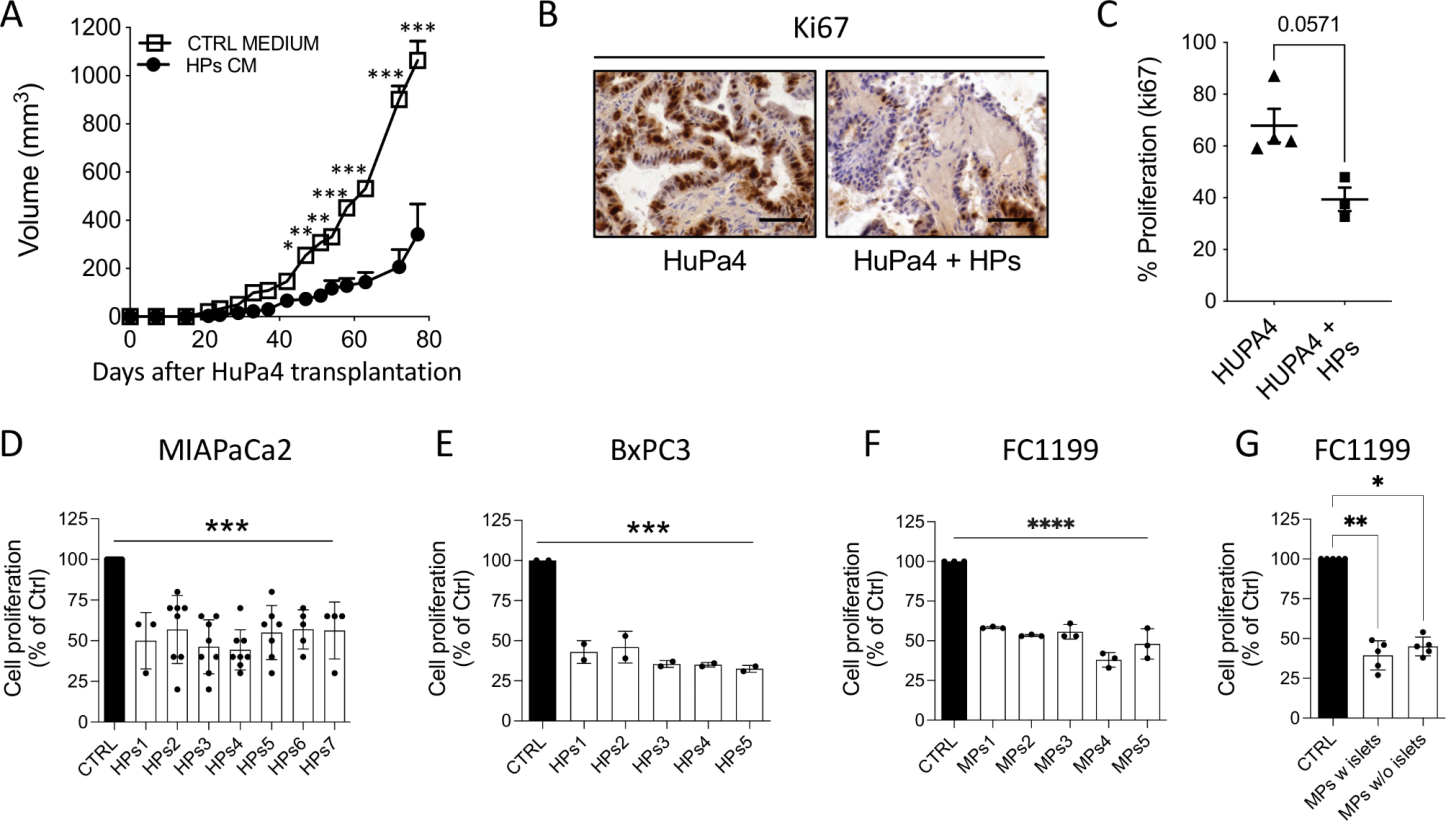
Effect of Ps conditioned media on PDAC growth. **(A)** Growth curve of HuPa4 tumors transplanted s.c and treated with HPs conditioned medium or control medium (mean ± SEM; n = 4 for each group, two-way ANOVA with Bonferroni’s multiple comparison test, *p < 0.005,**p < 0.0005, ***p < 0.0001). Ki67 immunostaining **(B)** and quantification of tumor cell proliferation **(C)** at euthanasia (400X, scale bar 100 μm) (mean ± SEM). Proliferation of MIAPaCa2 **(D)**, BxPC3 **(E)**, and FC1199 cells **(F)** in response to respectively 7 and 5 human and murine Ps CM, (72 h of treatment). Data are percentages of control from at least two different experiments (mean ± SEM; one-way ANOVA with Dunnett’s multiple comparison test, ***p < 0.001 ****p < 0.0001). **(G)** Proliferation of FC1199 cells in response to murine Ps CM, containing or not Langerhans islets. Data are percentages of control from at least five different Ps preparations (mean ± SEM; one-way ANOVA with Dunnett’s multiple comparison test, **p < 0.01, *p < 0.05)

To determine whether the inhibitory activity on tumors was mediated by released soluble factors we tested the effect of HP conditioned media (HPs CM) on in vivo growth of the HuPa4 PDX. Peritumoral injections of HPs CM significantly inhibited subcutaneous tumor growth (p < 0.0001), causing a 20-day delay in tumor onset and affecting the growth rate, with a lower percentage of Ki67^+^ proliferating tumor cells in the treated tumors compared with controls (Fig. 2A,B,C).

We then analyzed the effect of spheroid conditioned media in vitro on human MIAPaCa2 and BxPC3, as HuPa4 do not grow *in vitro.* Conditioned media from different preparations of HPs significantly inhibited MIAPaCa2 and BxPC3 cell proliferation (Fig. 2D and E). Similarly, CM from murine spheroids inhibited FC1199 cell proliferation (Fig. 2F).

To investigate whether the presence of the endocrine portion of the pancreas contributed to the inhibitory effect of spheroid-conditioned media, we prepared murine pancreatic spheroids containing or not the endocrine Langerhans islets. As depicted in Fig. 2G conditioned media of spheroids with or without Langerhans islets had the same inhibitory activity on FC1199 proliferation, confirming that the exocrine portion alone was responsible for inhibition.

**Fig. 3:**
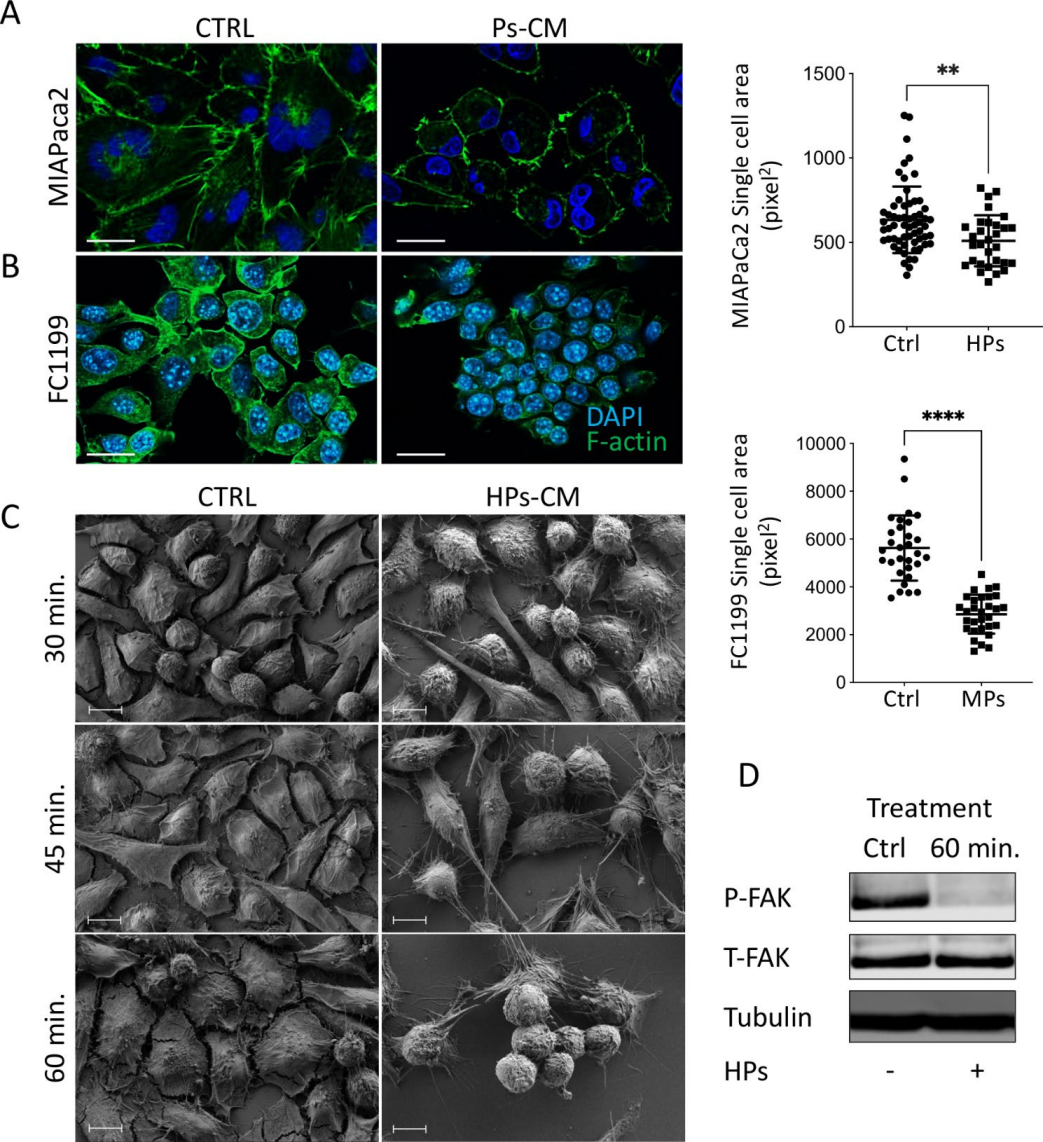
Ps-CM induce tumor cell detachment. Immunofluorescence analysis of F-actin (green) and quantification of single cell area of MIAPaCa2 **(A)** and FC1199 **(B)** cells after treatment with Ps CM (scale bar 25 μm) (mean ± SEM; Mann-Whitney, **p < 0.005,****p < 0.0001). **(C)** Scanning electron microscopy (SEM) of MIAPaCa2 cells after 30, 45 and 60 min of treatment with HPs CM (scale bar 10 μm). **(D)** Western Blot analysis of phospho-FAK, total-FAK, and tubulin of MIAPaCa2 cells after 60 min of treatment with HPs CM.

Inhibition of tumor growth was accompanied by striking rearrangement of the actin cytoskeleton and significant reduction in the MIAPaCa2 (Fig. 3A) and FC1199 (Fig. 3B) cell area. Scanning electron microscopy (SEM) analysis indicated rapid, progressive tumor cell detachment associated with rounded morphology in cells treated with HPs CM. Already after 30 min MIAPaCa2 cells showed changes in cell shape with extensions and thin projections. Cell rounding increased progressively with time and after 60 min cells were connected to the substrate only by cellular protrusions (Fig. 3C). Consistent with the effects on cell adhesion, Western blot analysis showed down-regulation of focal adhesion kinase (FAK) phosphorylation after 60 min of treatment (Fig. 3D) [16].

### HPs conditioned media inhibit FAK and FGFR signaling

**Fig. 4:**
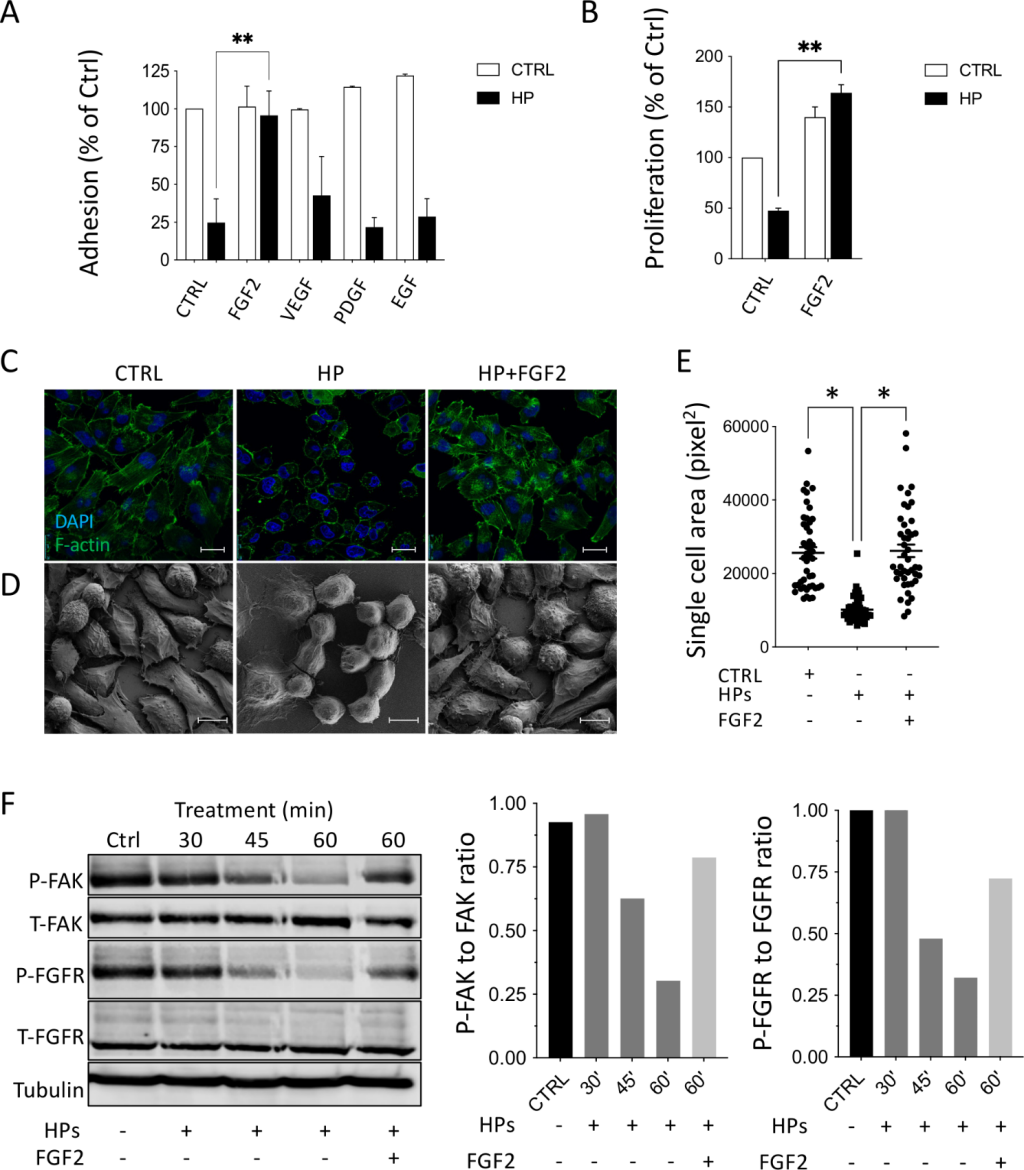
FGF2 blocks the de-adhesive effect of HPs CM on MIAPaCa2 cells. MIAPaCa2 cells were treated with HPs CM together with the growth factors (20 ng/mL). **(A)** Quantification of cell adhesion after 1 h of treatment (mean ± SEM; one-way ANOVA with Dunnett’s multiple comparison test, **p < 0.05). **(B)** Quantification of cell proliferation after 72 h of treatment (mean ± SEM; one-way ANOVA with Dunnett’s multiple comparison test, **p < 0.005). **(C)** Immunofluorescence analysis of F-actin (green, scale bar 25 μm) and **(D)** Scanning electron microscopy (SEM) (scale bar 10 μm) after 1 h of treatment with HPs CM with or without FGF (20 ng/mL). **(E)** Analysis of single cell area after 1 h of treatment (mean ± SEM; one-way ANOVA with Dunnett’s multiple comparison test, *p < 0.001). **(F)** Western Blot analysis of P-FAK, T-FAK, P-FGFR, T-FGFR and tubulin in MIAPaCa2 cell lysates after treatments and quantification of FAK and FGFR phosphorylation expressed as the ratio of phosphorylated protein to total protein

We next analyzed whether growth factors (FGF2, VEGF, PDGF, EGF) affecting FAK phosphorylation [17], and present in the PDAC microenvironment could reverse the activity of HPs. Among the growth factors tested, only FGF2 restored tumor cells adhesion (Fig. 4A,C-E) and proliferation (Fig. 4B) to levels comparable to controls or to FGF2 in the absence of HPs CM. This indicates a functional interaction between FGFR proliferative signaling and cell adhesive pathways.

Inhibition of FGFR1 phosphorylation paralleled the inhibition of FAK phosphorylation in MIAPaCa2 cells. HPs CM gradually reduced the phosphorylation of both FGFR1 and FAK after 45 and 60 min of treatment. The addition of FGF2 reversed both FGFR1 and FAK dephosphorylation, corroborating the hypothesis of a communication between the two signaling pathways. FAK and FGFR expression were not affected by the treatment (Fig. 4F).

### FGFR and α5β1 mediate the effect of HPs conditioned medium on PDAC cell adhesion

**Fig. 5:**
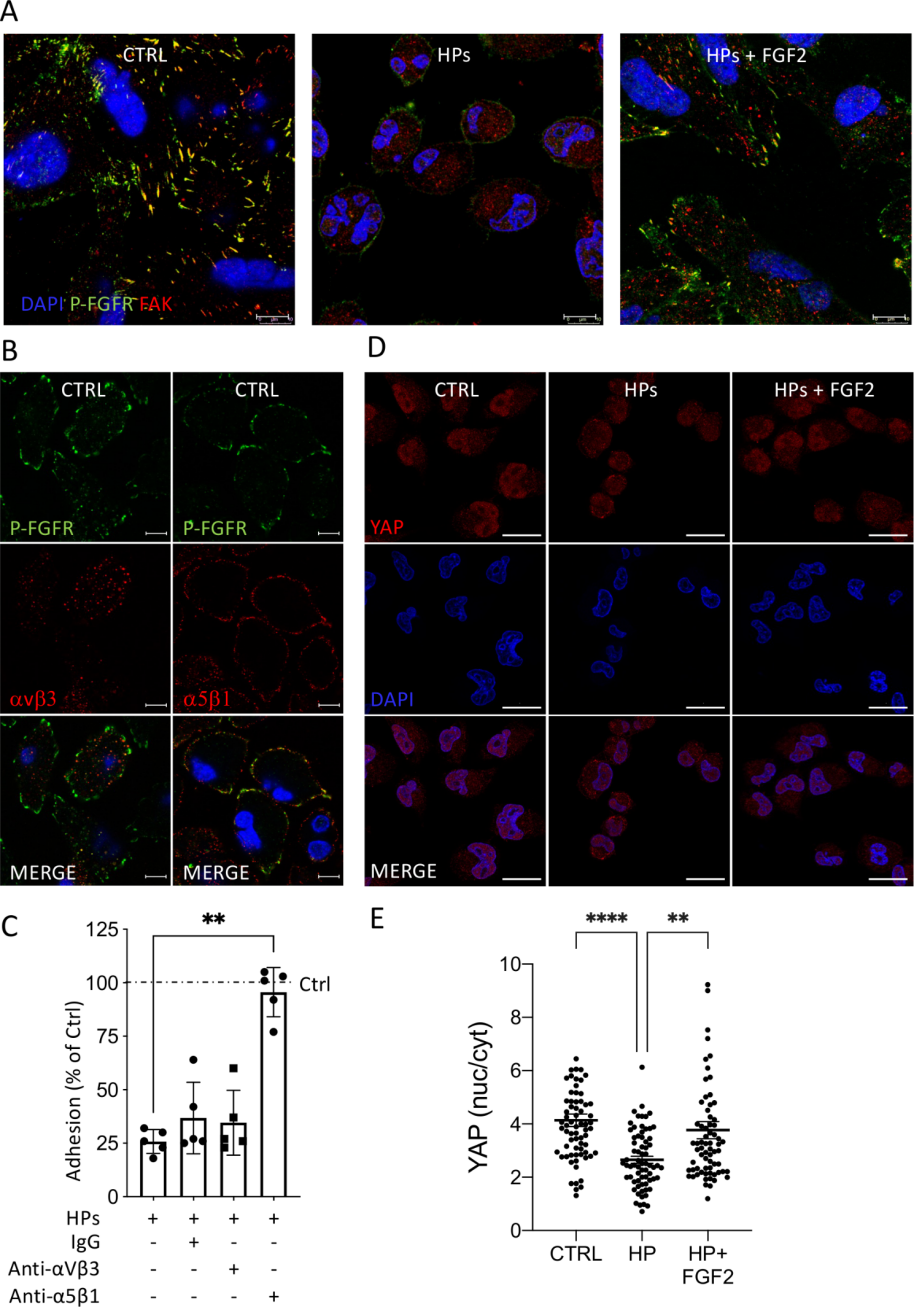
HPs CM disrupts P-FGFR, FAK and integrin α5β1 co-localization and induces YAP nuclear de-localization in MIAPaCa2 cells. **(A)** Immunofluorescence analysis of P-FGFR (green) and FAK (red) in MIAPaCa2 cells after 1 h of treatment with HPs CM ± 20 ng/mL of FGF2 (scale bar 10 μm). **(B)** Immunofluorescence of P-FGFR (green), integrin αvβ3 (red) and integrin α5β1 (red) in MIAPaCa2 cells (scale bar 10 μm). **(C)** Effect of anti-integrin αvβ3 and anti-integrin α5β1 antibodies (20 µg/mL) on adhesion of MIAPaCa2 cells treated with HPs CM (mean ± SEM, one-way ANOVA with Dunnett’s multiple comparison test, **p < 0.0001). **(D)** Immunofluorescence of YAP in MIAPaCa2 cells after 2 h of treatment with HPs CM ± 10 ng/mL of FGF2 (scale bar 25 μm). **(E)** Quantification of YAP localization (nucleus/cytoplasm ratio) (mean ± SEM; one-way ANOVA with Dunnett’s multiple comparison test, **p < 0.005,****p < 0.0001)

Immunofluorescence and confocal microscopy analysis showed that P-FGFR and FAK co-localize in the tips of MIAPaCa2 cells. Co-localization was disrupted by a 1 h-treatment with HPs CM, with FAK redistributing diffusely in the cells (Fig. 5A). The disruption was overcome by addition of FGF2.

Integrins interact with growth factor tyrosine kinase receptors [18] influencing FAK signaling. MIAPaCa2 expressed both α5β1 and αvβ3, but only α5β1 co-localized with P-FGFR at focal contacts (Fig. 5B). Antibodies anti-α5β1, but not anti-αvβ3, prevented MIAPaCa2 cell detachment induced by HPs CM (Fig. 5C) indicating the role of integrin-α5β1 in cooperation with FGFR1 in the effect of HPs CM on PDAC cell adhesion.

The transcription factor YAP mediates cell responses to the microenvironment [19]. HPs CM treatment of MIAPaCa2 cells caused YAP translocation from the nucleus to the cytoplasm. This effect was reversed by FGF2 suggesting the involvement of YAP, downstream FGFR and FAK in this system (Fig. 5D and E).

### Fibronectin proteolytic fragments generated by trypsin inhibits PDAC cell proliferation

Fibronectin (FN) mediates different interactions between cells and extracellular molecules, regulating cell adhesion and proliferation. It has numerous binding domains for integrins, growth factors and extracellular matrix components that can be exposed by conformational changes or proteolytic cleavage [20]. FN fragments generated by proteolysis have different and sometimes opposing biological effects [21]. Since FN is one of the main constituent of PDAC stroma involved in extracellular matrix remodeling and fibrosis [2], we investigated the presence and role of FN in HPs CM.

**Fig. 6:**
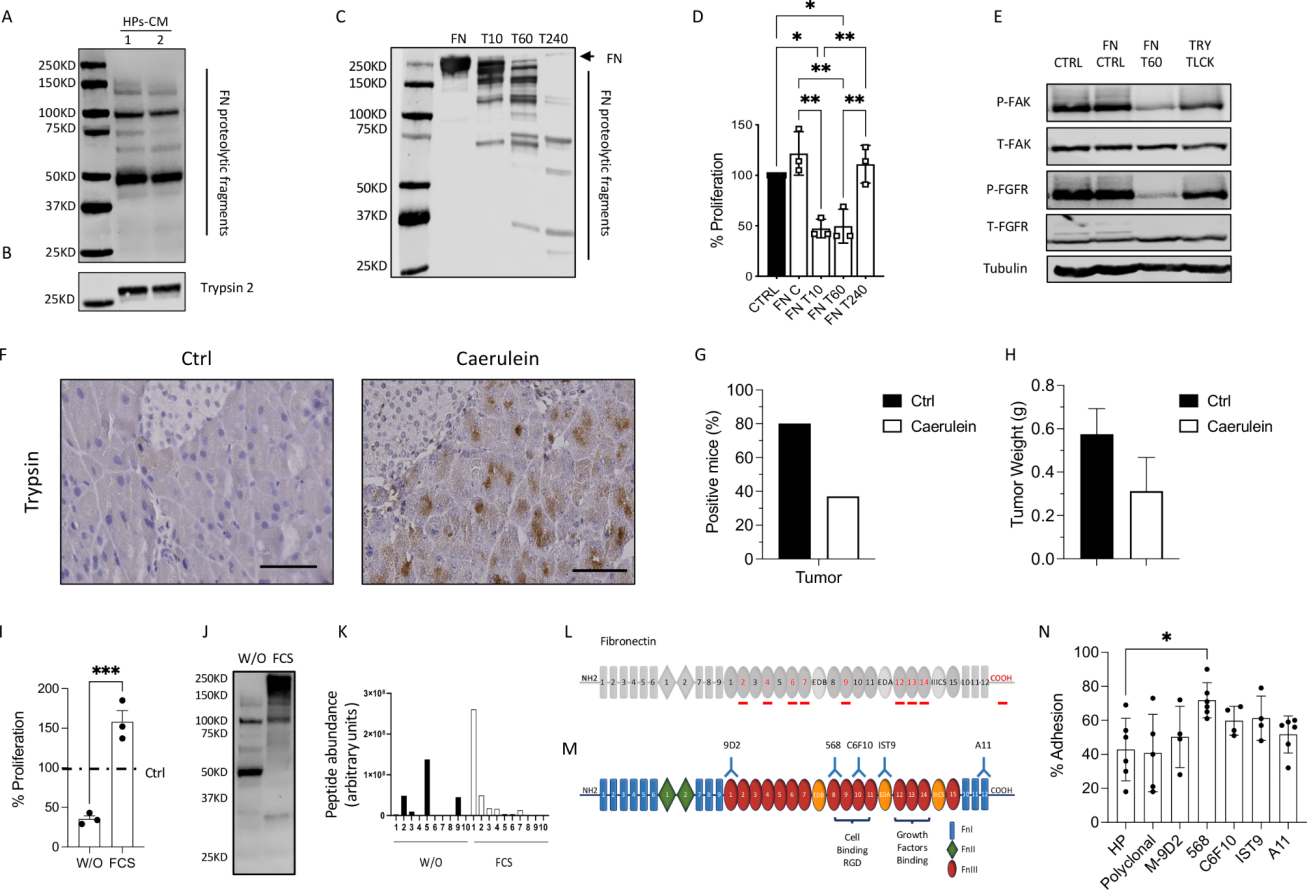
Fibronectin fragments induce PDAC cell detachment and FAK and FGFR dephosphorylation. **(A)** Western Blot analysis of fibronectin fragments and **(B)** trypsin in HPc CM. **(C)** Western Blot analysis of fibronectin fragments generated by trypsin after 10, 60 and 240 min of digestion (1:50, w/w). **(D)** Proliferation of MIAPaCa2 cells in response to control fibronectin (FN C), fibronectin digested for 10, 60, 240 min (FN T10, T60, T240). Data are percentages of control from at least three different experiments (mean ± SEM; one-way ANOVA with Dunnett’s multiple comparison test, *p < 0.05, **p < 0.005). **(E)** Western Blot analysis of P-FAK, P-FGFR and tubulin in MIAPaCa2 treated with control medium, 50 µg/mL of fibronectin (FN CTRL), FN digested for 60 min with trypsin (FN T60) and control of trypsin and TLCK (TRY + TLCK). **(F)** Trypsin immuno-staining of mice pancreas with Caerulein induced acute pancreatitis (400X, scale bar 50 μm). **(G,H)** Growth of MIAPaCa2 tumors implanted 24 h post acute pancreatitis induction. Data are the percentage of mice positive to tumor in comparison to ctrl mice (G) and tumor weight (H). **(I)** Proliferation of MIAPaCa2 cells in response to media conditioned by inhibitory (W/O) and activated (FCS) spheroids. Data are percentages of control (dotted line) from at least three different experiments (mean ± SEM; one-way ANOVA with Tukey’s multiple comparison test, ***p = 0.0001). **(J)** Western blot analysis of FN fragments in media conditioned by inhibitory (W/O) and activated (FCS) spheroids. **(K)** FN peptide abundance in each gel slice in media conditioned by inhibitory (W/O) and activated (FCS) spheroids was obtained by label-free mass spectrometry-based proteomics analysis. Peptide abundance is reported in arbitrary units. **(L)** Red lines highlights the identified peptides in media conditioned by inhibitory spheroids. **(M)** FN consists of two similar monomers of about 250 KDa linked by a disulfide bond at the C-terminus. Schematic representation of FN (monomer) and its interaction with anti-FN mAb used in adhesion experiments. **(N)** Effect of anti-FN mAb (20 µg/mL) in combination with HPs CM on MIAPaCa2 cell adhesion (mean ± SEM, one-way ANOVA with Dunnett’s multiple comparison test, *p < 0.05)

Western blot analysis of two HPs CM using polyclonal Ab showed multiple FN fragments (Fig. 6A). The CM also contained trypsin 2, the main proteolytic enzyme produced by the exocrine pancreas capable of generating FN proteolytic fragments (Fig. 6B).

We investigated whether the FN fragments generated by trypsin were active in our system. Digestion of human plasma FN with trypsin generated fragments that significantly inhibited tumor cell proliferation. Notably, only fragments generated after 10 and 60 min were active, whereas smaller fragments generated by further degradation were not active (Fig. 6C and D). The FN fragments generated by 60 min of trypsin treatment inhibited FAK and FGFR phosphorylation and the effect was reversed by the trypsin inhibitor TLCK (Fig. 6E).

To test the role of trypsin in our system, mouse pancreatic spheroids (MPs) were cultured with or without the trypsin inhibitors aprotinin or TLCK. At a concentration that inhibited trypsin enzymatic activity (Supplementary Fig. 2A), both aprotinin and TLCK reduced the activity of MPs CM on PDAC cell proliferation and FAK and FGFR phosphorylation (Supplementary Fig. 2B and C). Aprotinin or TLCK added directly to tumor cells with MPs CM did not prevent the conditioned medium’s inhibitory activity, confirming that MIAPaCa2 cell inhibition was not due to a direct effect of trypsin on tumor cells (data not shown).

Premature trypsin activation plays a pivotal role in human acute pancreatitis [22]. The presence of active trypsin in the exocrine spheroids might indicate that these spheroids reproduce a condition of damaged pancreas and suggests that a situation of acute pancreatitis might contrast tumor growth. We then investigated the effect of experimental pancreatitis on MIAPaCa2 in vivo growth. Repeated intraperitoneal administrations of caerulein induced acute pancreatitis [23], with high levels of plasmatic lipase and amylase. Acute pancreatitis was accompanied by intra-acinar trypsin activation and resulted in a significant inhibitory effect on MIAPaCa2 tumor growth, as observed with pancreatic spheroids (Fig. 6F,G,H and Supplementary Fig. 3).

### Identification of FN fragments responsible for tumor growth inhibition

The addition of platelet-derived factors or FCS to culturing conditions caused a switch of MP to an activated phenotype [24, 25]. FCS-treated spheroids lost their inhibitory activity and became able to promote PDAC cell proliferation (Fig. 6I). This activation was associated with the presence of a different pattern of FN fragments (Fig. 6J). Mass spectrometry-based proteomics (MS) revealed a different abundance of FN unique peptides with different molecular weights in inhibitory and stimulatory CM. The inhibitory CM (w/o) had a high abundance of FN peptides in the gel slice number 5 at ~ 50–75 KDa (Fig. 6K and Supplementary Fig. 4). Surprisingly, when matched against the FN sequence, peptides in the gel slice 5 corresponded to a FN fragment of ~ 150KDa, covering the Type III domains and the c-terminal region (Fig. 6L). These results suggest the possible presence in slice 5 of two FN fragments with similar MW: ~ 50–75 KDa. Undigested FN (gel slice 1) was identified only in the activated non-inhibitory spheroid CM, consistent with the involvement of proteolytic fragmentation of FN in the CM inhibitory activity.

Of a panel of six monoclonal Abs reacting with different epitopes of FN (Fig. 6M) only Ab 568 recognizing the type III-8 domain, reversed the effect of HPs CM on MIAPaCa2 cell adhesion (Fig. 6N), suggesting the involvement of this domain in cell detachment. C6F10 and IST9 Abs partially reversed HPs CM effect indicating a minor contribution of other FN domains to the CM inhibitory activity.

### Pharmacological inhibition of FAK and FGFR reduces PDAC growth in vitro and in vivo

**Fig. 7:**
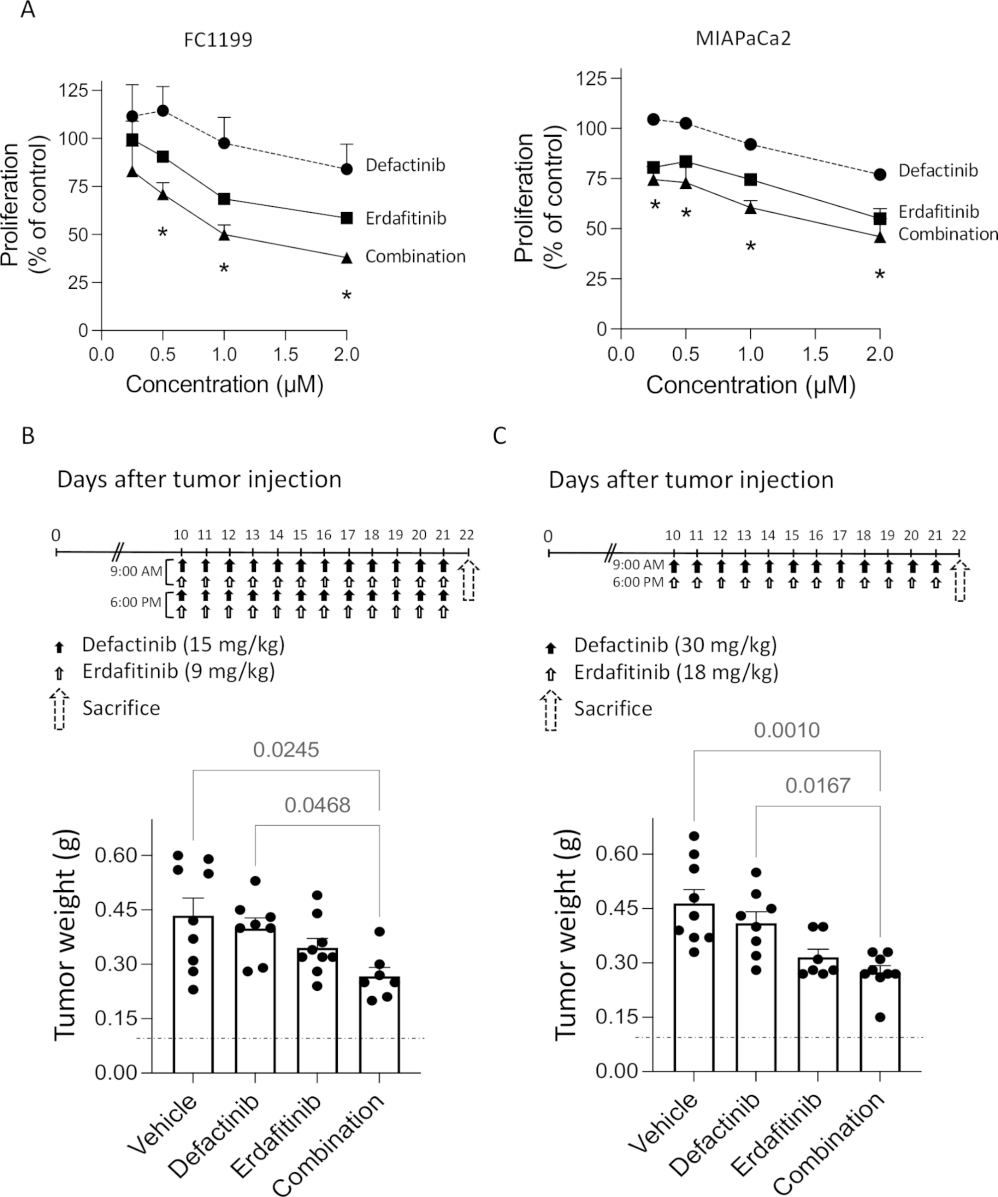
Defactinib and Erdafitinib inhibit FC1199 growth. **(A)***In vitro.* FC1199 proliferation in response to Defactinib (circles) and Erdafitinib (squares) singly or combined (triangles) (mean ± SEM of two experiments, two-way ANOVA with multiple comparison test, *p < 0.005). *In vivo.* Tumor burden (pancreas weight) in mice bearing FC1199 orthotopic tumors, treated with vehicle, Defactinib [15 mg/kg], Erdafitinib [9 mg/kg] or the combination twice/day **(B)**; or vehicle, Defactinib [30 mg/kg], Erdafitinib [18 mg/kg] or the combination once/day **(C)** for 12 days. Dotted line: healthy pancreas (mean ± SEM, One-way ANOVA followed by Bonferroni)

Our results indicates that FAK and FGFR work in concert in PDAC growth. To assess the translational value of combining FAK and FGFR inhibitors in PDAC treatment, we examined the effect of the FAK inhibitor VS-6063 (Defactinib) on the growth of tumor cells in vitro and in vivo, in combination with Erdafitinib, a pan-FGFR inhibitor. In vitro, Defactinib and Erdafitinib together had additive inhibitory action on FC1199 and MIAPaCa2 cell proliferation (p < 0.005) (Fig. 7A). In vivo the activity of Defactinib and Erdafitinib was tested on FC1199 orthotopically growing tumors. Treatments with the two drugs started on day 10 after tumor cell injection, when tumors were detectable [9] with two different doses and schedules [26, 27]. Only tumors treated with Defactinib plus Erdafitinib (both treatment schedules), were significantly inhibited compared to controls. (Fig. 7B and C). The combination of the two drugs had considerable effects on the tumor stroma, significantly inhibiting fibrosis (Sirius red staining) and angiogenesis (CD31) (Supplementary Fig. 5) and inducing a non statistically significant increase in tumor cell death (cleaved Caspase3). A slight increase in cell proliferation (ki67) was also observed, possibly as a rebound effect after treatment (Supplementary Fig. 5).

## Discussion

This study demonstrates that FN fragments generated by pancreatic trypsin inhibit PDAC cell adhesion, proliferation and in vivo tumor growth by inhibiting the functional connections among β1 integrins, FAK and FGFR, hence contributing to pancreatic homeostasis and limiting PDAC progression. Our findings also support the use of FAK inhibitors together with FGFR TKI for PDAC treatment.

Several studies reported that stromal and epithelial components surrounding neoplastic cells can positively or negatively influence tumor growth [2, 14, 28, 29]. However, to the best of our knowledge, this is the first study in which 3D spheroids isolated from the pancreas have been used to study the effect of the pancreatic microenvironment on PDAC growth. This system reproduces the complex interactions among cells composing the pancreas, maintains the ratio between cell populations and cell interaction is not affected by exogenous extracellular matrix or synthetic scaffolds. Of note, spheroids also survive in vivo, transplanted orthotopically in mice, where they survive for at least four weeks ensuring a long-lasting effect on the co-transplanted tumors.

The finding that the spheroids release active trypsin, normally absent in healthy pancreas, indicates that, to some extent, the spheroids reproduce a damaged environment, and might be considered a model of damaged pancreas. In agreement, we found that experimental pancreatitis was able to inhibit PDAC growth. Other studies have reported that that trypsin in vitro can exert antitumor activity on different tumor cell lines by suppressing the EMT program and promoting cancer cell differentiation [30, 31]. A combination of trypsinogen and chymotrypsinogen inhibited tumor growth, invasion and angiogenesis both in vitro and in vivo and demonstrated clinical efficacy on a cohort of 46 patients with advanced tumors of different origin [32, 33]. Nevertheless, because of its complex activities, trypsin is not suitable for therapeutic purposes, and its proteolytic activity has to be tightly regulated to avoid tissue damage [34], inflammation, and even chronic pancreatitis and consequently PDAC [6, 7]. Instead, this study points to specific pathways downstream trypsin, specifically the FGFR and FAK signalling pathways as valid therapeutic targets. Our in vivo preclinical studies do in fact confirm the feasibility and efficacy of this approach.

Due to the large number of trypsin substrates, the role of trypsin in cancer is complex, depending on the molecular context and the signaling pathway directly or indirectly modulated in the target cells [8, 35–37]. This study indicates that pancreatic trypsin, besides its digestive functions, seems necessary to maintain pancreatic tissue homeostasis and counteract tumor growth. By generating FN fragments with antitumor activity trypsin has a protective effect at the site of primary tumor growth affecting FGFR and FAK tumor signaling.

Assembly of FN into a three-dimensional network is essential for maintaining tissue architecture and to regulate cell adhesion [38]. We speculate that trypsin-generated FN fragments initially restrain tumor progression in the pancreas. Then, when FN secretion and deposition by activated stroma and tumor cells exceed trypsin’s proteolytic capacity for generating FN fragments with antitumor activity, tumor cells spread and proliferate on intact FN scaffolds, escape normal environmental surveillance, and progress towards a more malignant phenotype. It has in fact been demonstrated that an intact FN matrix is vital for cell adhesion [39], and that proteolysis is an important mechanism to control FN turnover and assembly [40–42]. The functional importance of this is supported by our findings that proteolytic FN fragments were identified only in the CM of spheroids with tumor restraining activity, whereas high MW, intact FN molecules were found in the CM of spheroids with pro-tumorigenic activity. Additional studies are needed to validate the importance of these pathways in the complex tumor-microenvironment interactions in in vivo experimental and clinical settings.

The connection between trypsin activity, FN fragment generation and the effect of spheroid CM on tumor cell adhesion/proliferation is further supported by the fact that spheroids treated with FCS lost their inhibitory activity and did not generate the FN fragments because of the copious trypsin inhibitors in serum.

Plasma and cellular FN have distinct structural (splice isoforms) and functional properties. In our study, trypsin-generated FN fragments from both cellular (spheroids) and plasma (purified) FN had the same inhibitory effect on PDAC, indicating that the FN fragment responsible for these effects belongs to a domain conserved in the two forms [5, 43]. The FN type III-8 domain identified in medium conditioned by pancreatic spheroids with inhibitory activity is present in most FN isoforms. Indeed in some physiological and pathological processes it has been suggested that plasma and cellular FN perform the same functions, with cellular FN compensating for plasma FN deficiency [44]. In other processes such as wound healing, plasma and cellular FN play distinct roles in the different phases of tissue repair [45].

This study establishes a direct cause-effect relationship between trypsin-generated FN fragments and the effect on FGFR and FAK phosphorylation and co-localization with integrin α5β1. Other proteases, such as MMP2 [46], MMP9 [47], elastase [48], TAT-2 [35], and granzyme [49] cleave FN and influence several cell functions including cell adhesion and migration.

FN fragments have been previously identified both locally in tissues and body fluids and suggested to regulate cell activities [50]. In agreement with our findings, a time course study [51] indicated that FN cleavage occurred rapidly proximal to the C-terminal residues containing the interdimeric disulfides bonds, near the N-terminus (between I5 and I6 in Fig. 6M), and near the C-terminus (between III14 and III15), followed by slower cleavages that yield multiple bands. Here proteomic analysis identified peptides in the ~ 80 KD fragment that encompassed a ~ 150 KDa region. Considering the discrepancy between the sequence coverage of FN (150 KDa) and the fragment molecular weight (80 KDa), we assumed the concomitant presence of two FN fragments of similar molecular weight. Using a panel of monoclonal antibodies, we identified the type III-8 domain as the putative active region within the type III domain of FN, but further analysis are required to define the active site more precisely.

The FN multimodular structure and cell- and tissue-specific isoforms [52] allow various different and multiple interactions with cell-surface receptors, growth factors and other ECM proteins [20, 45]. Our findings are in agreement with other studies supporting the existence of a complex interaction between FN-FN fragments, FGFR1 and integrins. FN transactivates FGFR1 through β1 integrin in liver endothelial cells [21]. FNIII 9–10 collaborates with FGFRs to promote neuronal cell adhesion [53]. Changes in integrin receptor affinity have been reported to be triggered by soluble rather than immobilized FN [54]. A direct interaction of FGF1 with integrin αvβ3 and FGFR has been demonstrated [55].

To our knowledge, our study is the first one to demonstrate that FN proteolytic fragments generated by pancreatic cells can reduce P-FGFR, P-FAK and α5β1 co-localization in PDAC cells.

FGFR and FAK pathways drive PDAC progression [56–58] and have been targeted separately in clinical and preclinical trials in combination with cytotoxic agents and immune-checkpoint inhibitors, but with limited efficacy [59, 60]. Our in vitro and in vivo results confirm the partial inhibitory activity of the single inhibitors, but also indicate that combining FGFR and FAK inhibitors results in additive tumor growth inhibition and therefore could be a valid therapeutic approach to be considered in the clinical setting.

## Conclusions

We demonstrate that FGFR1, integrin α5β1 and FAK are functionally interconnected and contribute to PDAC growth. By generating FN fragments with antitumor activity, pancreatic trypsin – possibly released during pancreas pathologies or tissue damage – has a protective effect against primary tumor growth, affecting FGFR and FAK tumor signaling. Targeting FGFR and FAK simultaneously may be an effective strategy to limit the aggressiveness of PDAC. Confirmatory clinical studies are required to validate such therapeutic approach.

### Electronic supplementary material

Below is the link to the electronic supplementary material.

Supplementary Material 1

Supplementary Material 2

Supplementary Material 3

Supplementary Material 4

Supplementary Material 5

Supplementary Material 6

Supplementary Material 7

## Data Availability

The mass spectrometry proteomics data have been deposited at the ProteomeXchange Consortium via the PRIDE partner repository [61] with the dataset identifier PXD035770.

## References

[1] Falcomatà C, Saur D (2021). Self-renewal equality in pancreas homeostasis, regeneration, and cancer. Cell Rep.

[2] Jiang H, Torphy RJ, Steiger K (2020). Pancreatic ductal adenocarcinoma progression is restrained by stromal matrix. J Clin Invest.

[3] Mahadevan D, Von Hoff DD (2007). Tumor-stroma interactions in pancreatic ductal adenocarcinoma. Mol Cancer Ther.

[4] Öhlund D, Handly-Santana A, Biffi G (2017). Distinct populations of inflammatory fibroblasts and myofibroblasts in pancreatic cancer. J Exp Med.

[5] López-Otín C, Bond JS (2008). Proteases: multifunctional enzymes in life and disease. J Biol Chem.

[6] Hirota M, Ohmuraya M, Baba H (2006). The role of trypsin, trypsin inhibitor, and trypsin receptor in the onset and aggravation of pancreatitis. J Gastroenterol.

[7] Ji B, Gaiser S, Chen X (2009). Intracellular trypsin induces pancreatic acinar cell death but not NF-kappaB activation. J Biol Chem.

[8] Yamashita K, Mimori K, Inoue H (2003). A tumor-suppressive role for trypsin in human cancer progression. Cancer Res.

[9] Resovi A, Borsotti P, Ceruti T (2020). CCN-Based therapeutic peptides modify pancreatic ductal adenocarcinoma microenvironment and decrease Tumor Growth in Combination with Chemotherapy. Cells.

[10] Dossena M, Piras R, Cherubini A (2020). Standardized GMP-compliant scalable production of human pancreas organoids. Stem Cell Res Ther.

[11] Workman P, Aboagye EO, Balkwill F (2010). Guidelines for the welfare and use of animals in cancer research. Br J Cancer.

[12] Resovi A, Bani MR, Porcu L (2018). Soluble stroma-related biomarkers of pancreatic cancer. EMBO Mol Med.

[13] Brunelli L, Llansola M, Felipo V (2012). Insight into the neuroproteomics effects of the food-contaminant non-dioxin like polychlorinated biphenyls. J Proteom.

[14] Biffi G, Tuveson DA (2021). Diversity and Biology of Cancer-Associated fibroblasts. Physiol Rev.

[15] Baldan J, Houbracken I, Rooman I (2019). Adult human pancreatic acinar cells dedifferentiate into an embryonic progenitor-like state in 3D suspension culture. Sci Rep.

[16] Fan Y, Qu X, Ma Y (2016). Cbl-b promotes cell detachment via ubiquitination of focal adhesion kinase. Oncol Lett.

[17] Hunger-Glaser I, Fan RS, Perez-Salazar E (2004). PDGF and FGF induce focal adhesion kinase (FAK) phosphorylation at Ser-910: dissociation from Tyr-397 phosphorylation and requirement for ERK activation. J Cell Physiol.

[18] Brown WS, Tan L, Smith A (2016). Covalent targeting of fibroblast growth factor receptor inhibits metastatic breast Cancer. Mol Cancer Ther.

[19] Zanconato F, Cordenonsi M, Piccolo S (2019). YAP and TAZ: a signalling hub of the tumour microenvironment. Nat Rev Cancer.

[20] Lin T-C, Yang C-H, Cheng L-H (2019). Fibronectin in Cancer: friend or foe. Cells.

[21] Zou L, Cao S, Kang N (2012). Fibronectin induces endothelial cell migration through β1 integrin and src-dependent phosphorylation of fibroblast growth factor receptor-1 at tyrosines 653/654 and 766. J Biol Chem.

[22] Whitcomb DC, Gorry MC, Preston RA (1996). Hereditary pancreatitis is caused by a mutation in the cationic trypsinogen gene. Nat Genet.

[23] Marui S, Nishikawa Y, Shiokawa M (2022). Context-dependent roles of Hes1 in the adult pancreas and pancreatic tumor formation. Gastroenterology.

[24] Samoszuk M, Tan J, Chorn G (2005). Clonogenic growth of human breast cancer cells co-cultured in direct contact with serum-activated fibroblasts. Breast Cancer Res BCR.

[25] Yan M, Jurasz P (2016). The role of platelets in the tumor microenvironment: from solid tumors to leukemia. Biochim Biophys Acta.

[26] Le Large TYS, Bijlsma MF, El Hassouni B (2021). Focal adhesion kinase inhibition synergizes with nab-paclitaxel to target pancreatic ductal adenocarcinoma. J Exp Clin Cancer Res.

[27] Perera TPS, Jovcheva E, Mevellec L (2017). Discovery and Pharmacological characterization of JNJ-42756493 (Erdafitinib), a functionally selective small-molecule FGFR family inhibitor. Mol Cancer Ther.

[28] Belotti D, Foglieni C, Resovi A (2011). Targeting angiogenesis with compounds from the extracellular matrix. Int J Biochem Cell Biol.

[29] Hynes RO (2009). The extracellular matrix: not just pretty fibrils. Science.

[30] Hernández-Camarero P, López-Ruiz E, Griñán-Lisón C (2019). Pancreatic (pro)enzymes treatment suppresses BXPC-3 pancreatic Cancer stem cell subpopulation and impairs tumour engrafting. Sci Rep.

[31] Perán M, Marchal JA, García MA (2013). In vitro treatment of carcinoma cell lines with pancreatic (pro)enzymes suppresses the EMT programme and promotes cell differentiation. Cell Oncol Dordr.

[32] González-Titos A, Hernández-Camarero P, Barungi S (2021). Trypsinogen and chymotrypsinogen: potent anti-tumor agents. Expert Opin Biol Ther.

[33] Perán M, López-Ruiz E, García M (2017). A formulation of pancreatic pro-enzymes provides potent anti-tumour efficacy: a pilot study focused on pancreatic and ovarian cancer. Sci Rep.

[34] Kukor Z, Tóth M, Pál G (2002). Human cationic trypsinogen. Arg(117) is the reactive site of an inhibitory surface loop that controls spontaneous zymogen activation. J Biol Chem.

[35] Koivunen E, Ristimäki A, Itkonen O (1991). Tumor-associated trypsin participates in cancer cell-mediated degradation of extracellular matrix. Cancer Res.

[36] Lahey KA, Ronaghan NJ, Shang J (2017). Signaling pathways induced by serine proteases to increase intestinal epithelial barrier function. PLoS ONE.

[37] Miike S, McWilliam AS, Kita H (2001). Trypsin induces activation and inflammatory mediator release from human Eosinophils through protease-activated Receptor-2. J Immunol.

[38] Chernousov MA, Fogerty FJ, Koteliansky VE (1991). Role of the I-9 and III-1 modules of fibronectin in formation of an extracellular fibronectin matrix. J Biol Chem.

[39] Sottile J, Hocking DC (2002). Fibronectin polymerization regulates the composition and stability of extracellular matrix fibrils and cell-matrix adhesions. Mol Biol Cell.

[40] Mosher DF, Fogerty FJ, Chernousov MA (1991). Assembly of fibronectin into extracellular matrix. Ann N Y Acad Sci.

[41] Schnellmann R, Sack R, Hess D (2018). A selective Extracellular Matrix Proteomics Approach identifies Fibronectin Proteolysis by a disintegrin-like and metalloprotease domain with Thrombospondin Type 1 Motifs (ADAMTS16) and its impact on spheroid morphogenesis. Mol Cell Proteomics MCP.

[42] Shi F, Sottile J (2011). MT1-MMP regulates the turnover and endocytosis of extracellular matrix fibronectin. J Cell Sci.

[43] Hoshijima M, Hattori T, Inoue M (2006). CT domain of CCN2/CTGF directly interacts with fibronectin and enhances cell adhesion of chondrocytes through integrin alpha5beta1. FEBS Lett.

[44] Sakai T, Johnson KJ, Murozono M (2001). Plasma fibronectin supports neuronal survival and reduces brain injury following transient focal cerebral ischemia but is not essential for skin-wound healing and hemostasis. Nat Med.

[45] To WS, Midwood KS (2011). Plasma and cellular fibronectin: distinct and independent functions during tissue repair. Fibrogenesis Tissue Repair.

[46] Steffensen B, Chen Z, Pal S (2011). Fragmentation of fibronectin by inherent autolytic and matrix metalloproteinase activities. Matrix Biol J Int Soc Matrix Biol.

[47] Kusubata M, Hirota A, Ebihara T (1999). Spatiotemporal changes of fibronectin, tenascin-C, fibulin-1, and fibulin-2 in the skin during the development of chronic contact dermatitis. J Invest Dermatol.

[48] Grinnell F, Zhu M (1996). Fibronectin degradation in chronic wounds depends on the relative levels of elastase, alpha1-proteinase inhibitor, and alpha2-macroglobulin. J Invest Dermatol.

[49] Hendel A, Granville DJ (2013). Granzyme B cleavage of fibronectin disrupts endothelial cell adhesion, migration and capillary tube formation. Matrix Biol J Int Soc Matrix Biol.

[50] Yi M, Ruoslahti E (2001). A fibronectin fragment inhibits tumor growth, angiogenesis, and metastasis. Proc Natl Acad Sci U S A.

[51] Smith DE, Mosher DF, Johnson RB (1982). Immunological identification of two sulfhydryl-containing fragments of human plasma fibronectin. J Biol Chem.

[52] Hershberger RP, Culp LA (1990). Cell-type-specific expression of alternatively spliced human fibronectin IIICS mRNAs. Mol Cell Biol.

[53] Choung PH, Seo BM, Chung CP (2002). Synergistic activity of fibronectin and fibroblast growth factor receptors on neuronal adhesion and neurite extension through extracellular signal-regulated kinase pathway. Biochem Biophys Res Commun.

[54] Faull RJ, Kovach NL, Harlan JM (1993). Affinity modulation of integrin alpha 5 beta 1: regulation of the functional response by soluble fibronectin. J Cell Biol.

[55] Mori S, Wu C-Y, Yamaji S (2008). Direct binding of integrin alphavbeta3 to FGF1 plays a role in FGF1 signaling. J Biol Chem.

[56] Carter EP, Coetzee AS, Tomas Bort E (2021). Dissecting FGF signalling to Target Cellular Crosstalk in Pancreatic Cancer. Cells.

[57] Jiang H, Hegde S, Knolhoff BL (2016). Targeting focal adhesion kinase renders pancreatic cancers responsive to checkpoint immunotherapy. Nat Med.

[58] Zhang H, Hylander BL, LeVea C (2014). Enhanced FGFR signalling predisposes pancreatic cancer to the effect of a potent FGFR inhibitor in preclinical models. Br J Cancer.

[59] Bejarano L, Jordāo MJC, Joyce JA (2021). Therapeutic targeting of the Tumor Microenvironment. Cancer Discov.

[60] Nevala-Plagemann C, Hidalgo M, Garrido-Laguna I (2020). From state-of-the-art treatments to novel therapies for advanced-stage pancreatic cancer. Nat Rev Clin Oncol.

[61] Perez-Riverol Y, Bai J, Bandla C (2022). The PRIDE database resources in 2022: a hub for mass spectrometry-based proteomics evidences. Nucleic Acids Res.

