# Supplementary figures and images for: Fibronectin fragments generated by pancreatic trypsin act as endogenous inhibitors of pancreatic tumor growth
Source: J Exp Clin Cancer Res. 2023 Aug 9;42:201. doi: 10.1186/s13046-023-02778-y (PMC10411016; doi:10.1186/s13046-023-02778-y)

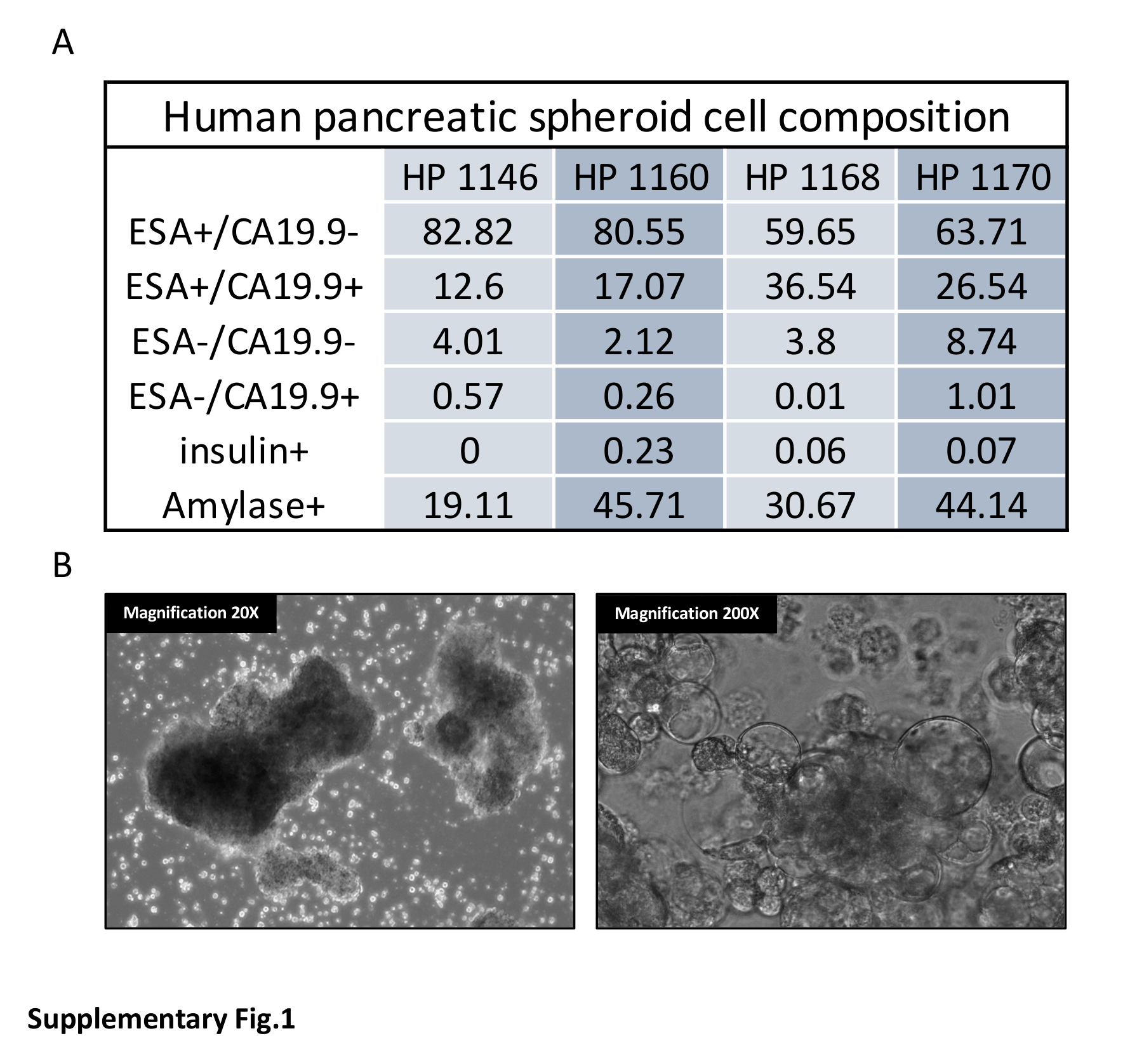

Supplement: Supplementary file 1 — Supplementary Material 1 [file 13046_2023_2778_MOESM1_ESM.tif]

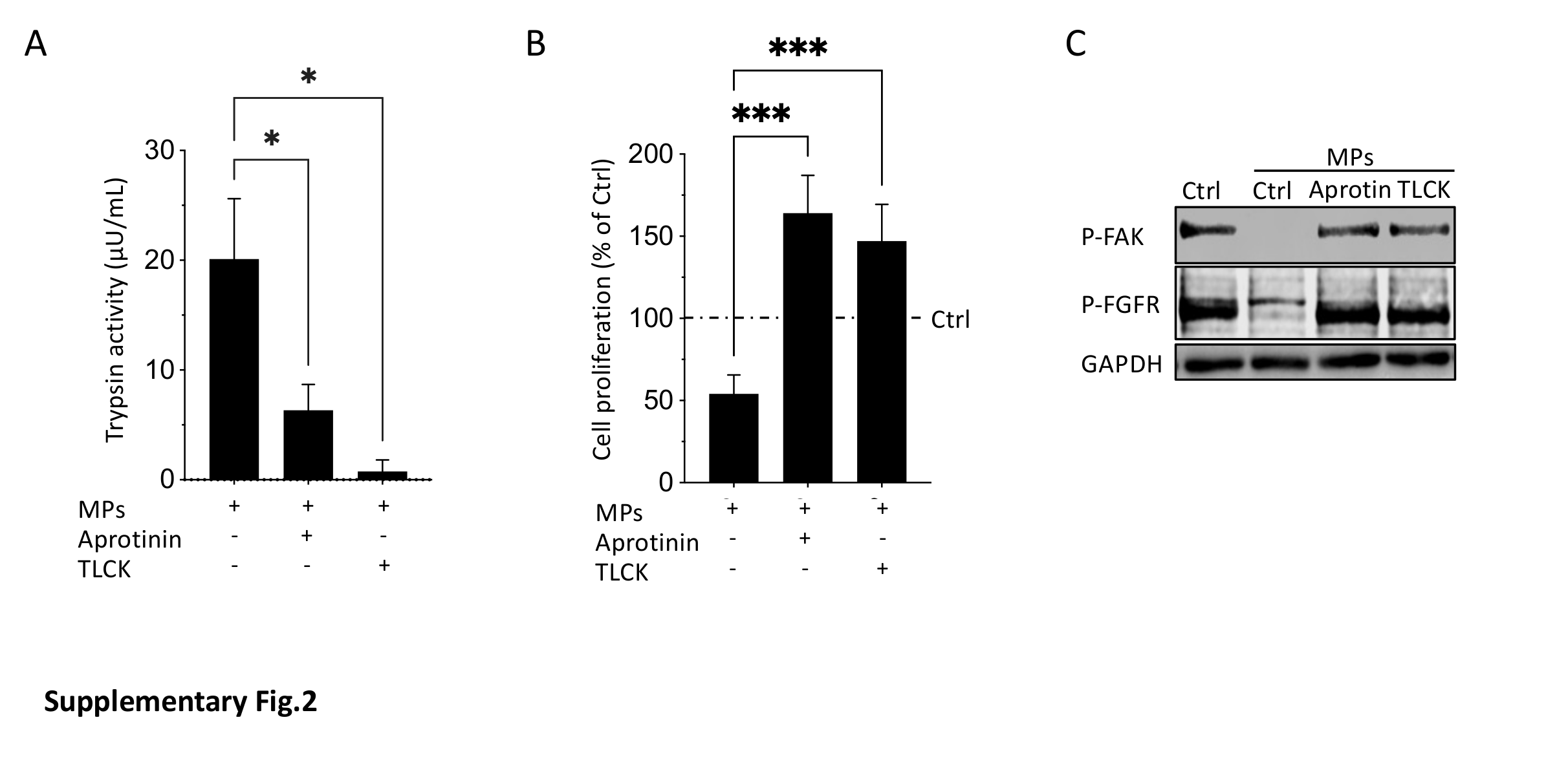

Supplement: Supplementary file 2 — Supplementary Material 2 [file 13046_2023_2778_MOESM2_ESM.tif]

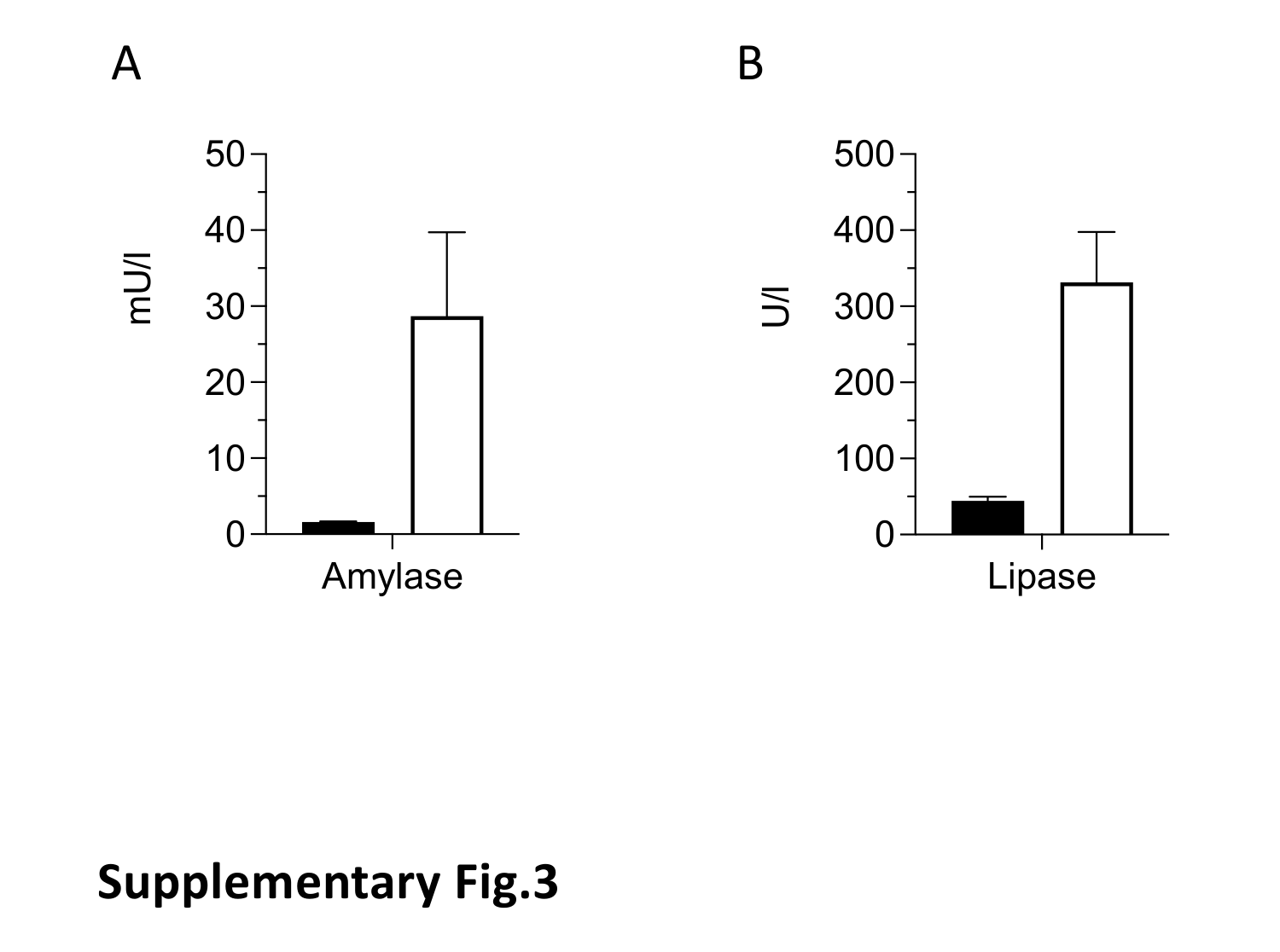

Supplement: Supplementary file 3 — Supplementary Material 3 [file 13046_2023_2778_MOESM3_ESM.tif]

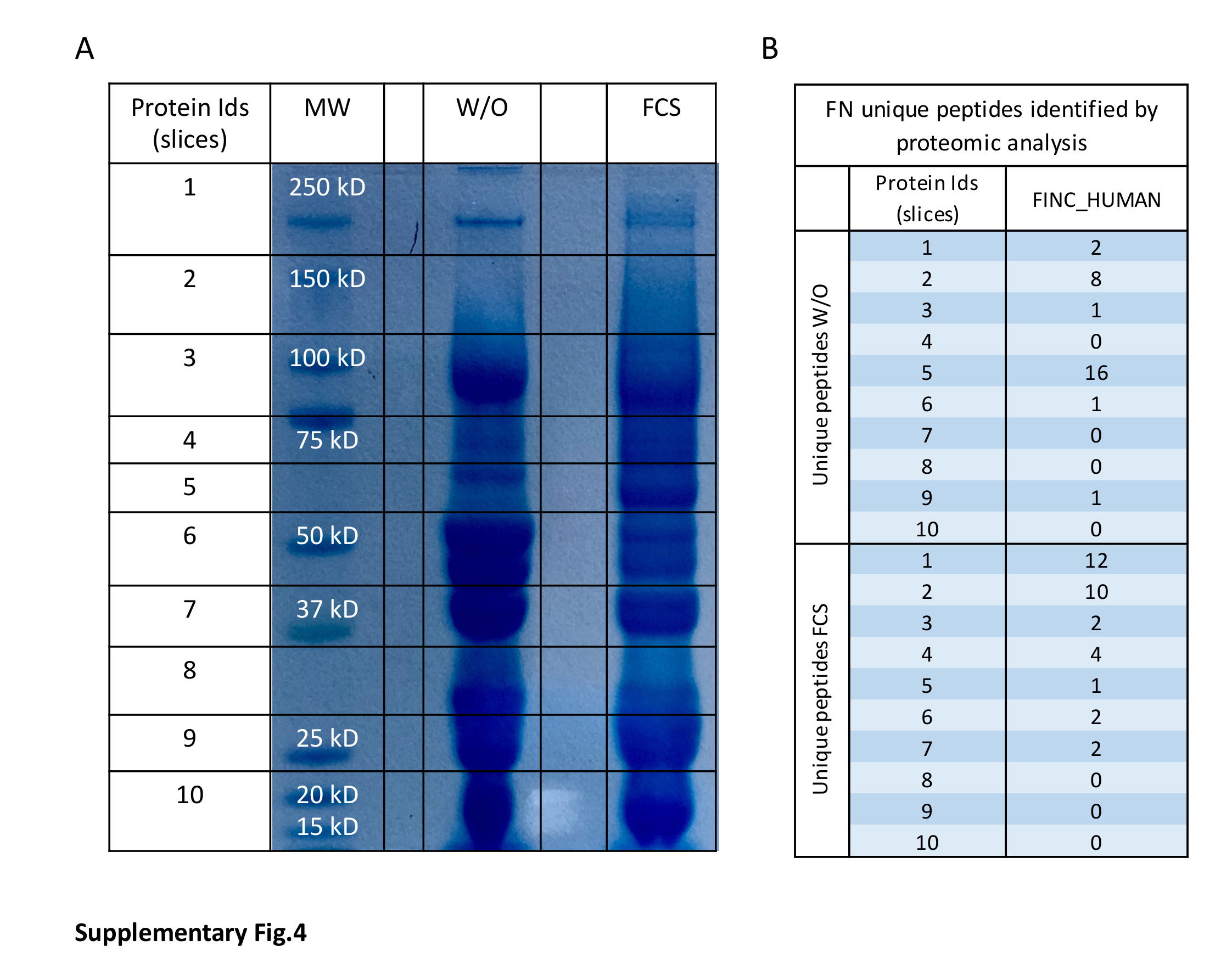

Supplement: Supplementary file 4 — Supplementary Material 4 [file 13046_2023_2778_MOESM4_ESM.tif]

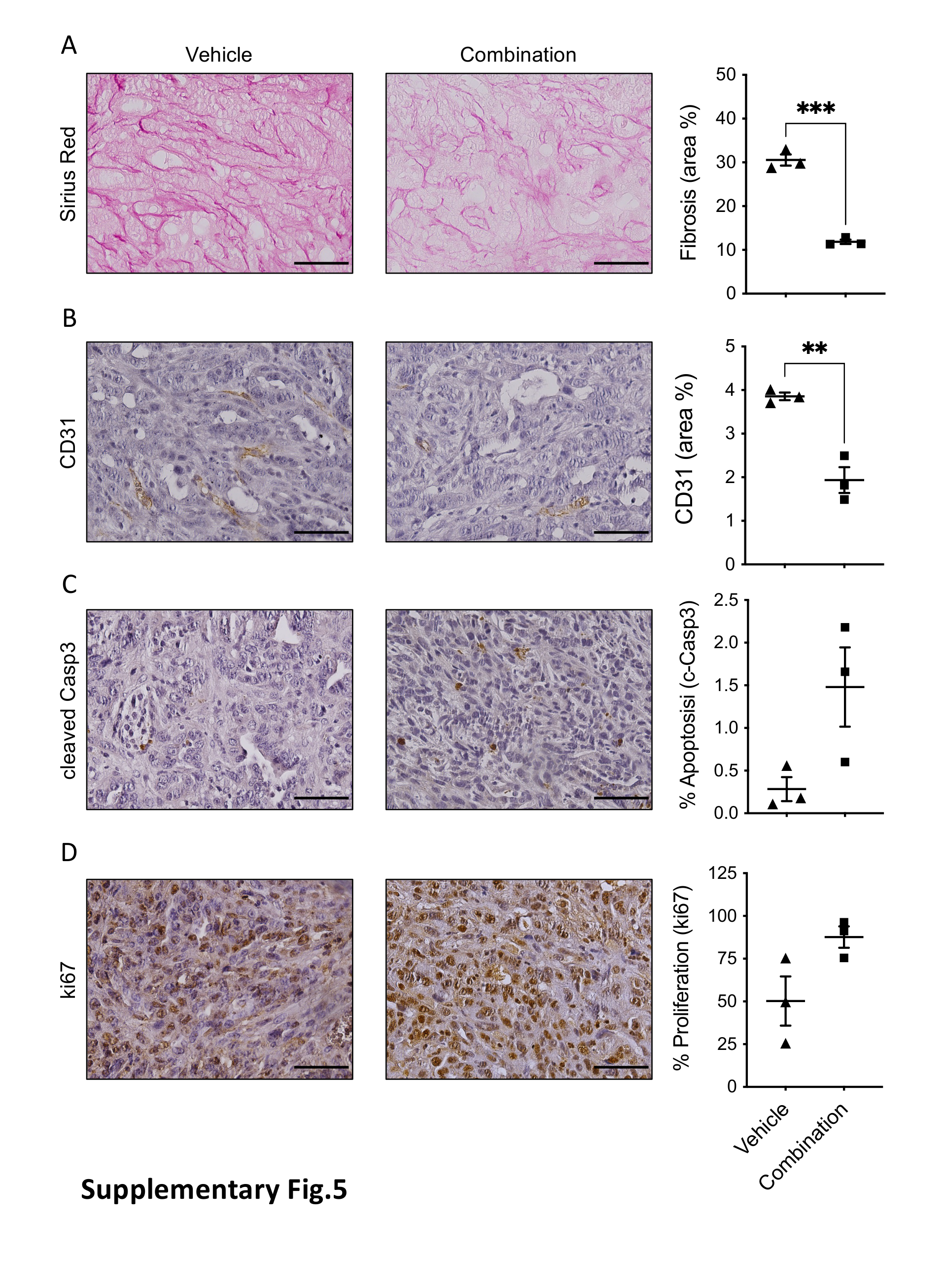

Supplement: Supplementary file 5 — Supplementary Material 5 [file 13046_2023_2778_MOESM5_ESM.tif]
