# Supplementary material for: Fibronectin fragments generated by pancreatic trypsin act as endogenous inhibitors of pancreatic tumor growth
Source: J Exp Clin Cancer Res. 2023 Aug 9;42:201. doi: 10.1186/s13046-023-02778-y (PMC10411016; doi:10.1186/s13046-023-02778-y)
Supplement: Supplementary file 6 — Supplementary Material 6 [file 13046_2023_2778_MOESM6_ESM.docx]

**Supplementary Fig.1 Human pancreatic spheroids.** (A) Cell composition (%) analyzed in four cell preparations and (B) in vitro growth as spheroid structures.

**Supplementary Fig.2 Aprotinin and TLCK abrogate MPs CM inhibitory activity.** MPs were treated with aprotinin (17 µg/mL) or TLCK (50 µg/mL). (A) Trypsin activity in MPs CM ± aprotinin or TLCK (mean ± SEM; one-way ANOVA with Dunnett’s multiple comparison test, *p<0.05). (B) Proliferation of MIAPaCa2 cells treated for 72 h with MPs CM ± aprotinin or TLCK. Data are percentages of control from at least three different experiments (mean ± SEM; one-way ANOVA with Tukey’s multiple comparison test, ***p<0.0001). (C) Western Blot analysis of P-FAK, P-FGFR and GAPDH in MIAPaCa2 cell lysates after 24 h of treatment with MPs CM ± aprotinin or TLCK.

**Supplementary Fig.3 Acute pancreatitis.** Plasmatic levels of amylase (A) and lipase (B).

**Supplementary Fig.4 Media conditioned by inhibitory (W/O) or activated (FCS) spheroids contain FN fragments of different MW.** (A) Equal amounts of proteins for each sample were separated by polyacrylamide gel electrophoresis and each gel lane was manually cut into 10 bands. (B) FN unique peptides in each gel slice, obtained by label-free mass spectrometry-based proteomics analysis.

**Supplementary Fig.5 Histological analysis of tumor treated with Defactinb and Erdafitinib in combination.** (A) Sirius red staining, immuno-staining of (B) CD31, (C) Cleaved caspase 3, (D) ki67 and their quantification at euthanasia (400X, scale bar 50 µm) (mean ± SEM; 3 for each group, Mann-Whitney, **p<0.005,***p<0.0005).
