# Supplementary material for: Fibronectin fragments generated by pancreatic trypsin act as endogenous inhibitors of pancreatic tumor growth
Source: J Exp Clin Cancer Res. 2023 Aug 9;42:201. doi: 10.1186/s13046-023-02778-y (PMC10411016; doi:10.1186/s13046-023-02778-y)
Supplement: Supplementary file 7 — Supplementary Material 7 [file 13046_2023_2778_MOESM7_ESM.docx]

**Histological and immunohistochemical analysis**

Tumors and tissues were collected, fixed in 10% phosphate-buffered formalin and embedded in paraffin; 4 μm-thick serial sections, were stained with H&E or by immunohistochemistry or immunofluorescence with Abs anti-human HLA-A 1:500 (abcam), anti-vimentin sp20 1:500 (ThermoFisher), anti-cytokeratin 1:1000 (Dako), anti-ki67 sp6 1:500 (ThermoFisher), anti-cleaved caspase-3 1:200 (Cell signaling, EuroClone), anti-trypsin 1:50 (Santa Cruz Biotechnology, Heidelberg, Germany), anti-CD31 1:50 (Dianova GmbH, Hamburg, Germany).

**Antibodies for immunofluorescence**

Primary: rabbit polyclonal anti-P-FGFR1 Tyr653/654 1:50 (Cell Signalling); mouse monoclonal anti-FAK 1:100 (abcam); mouse monoclonal anti-α5β1 1:100 (Chemicon, ThermoFisher); mouse monoclonal anti-αvβ3 1:100 (Chemicon); rabbit monoclonal anti-YAP 1:100 (Cell Signalling).

Secondary: goat Cy3 anti-mouse 1:50; goat FITC anti-rabbit 1:25 (Jackson ImmunoResearch Laboratories, EuroClone).

**Antibodies for WB**

Primary: rabbit polyclonal anti-P-FGFR1 Tyr653/654 1:1000 (Cell Signalling); mouse monoclonal anti-FAK 1:2000 (abcam); rabbit monoclonal anti-P-FAK 1:1000 (abcam); mouse monoclonal anti-trypsin 1:250 (Santa Cruz Biotechnology); rabbit polyclonal anti-fibronectin 1:1000 (abcam).

Secondary: anti-mouse per-ox, 1:10,000, anti-rabbit per-ox 1:50,000 (Sigma-Aldrich); IRDye 800CW goat anti-mouse IgG 1:5000 and IRDye 680RD goat anti-rabbit IgG 1:5000 (LI-CORE Biosciences GmbH, Bad Homburg, Germany).

**Scanning Electron Microscopy (SEM)**

Cells were fixed in 0.5% glutaraldehyde (Sigma Aldrich, St. Louis, MO, USA) for 1 h at 4°C, washed in cacodylate buffer, and post-fixed with 1% OsO_4_ (Società Italiana Chimici, Rome, Italy) for another hour. Fixed specimens were then dehydrated through a series of passages in increasing ethanol baths and dried in pure hexamethyldisilazane (HMDS, Fluka Chemie AG, Buchs, Switzerland). Samples were mounted on stubs, and coated with gold in a sputter coater (Agar Scientific Ltd, Stansted, England). Coated specimens were observed on a Cross-Beam 1540EsB electron microscope, using secondary electron detection (Supra55, Carl Zeiss GmbH, Germany).

**Trypsin activity assay**

The enzymatic activity of trypsin conditioned media was measured using a commercial colorimetric kit (Trypsin activity assay kit, abcam). Tests were run according to the manufacturer’s directions.

**Proteomics**

Equal amounts of proteins for each sample (inhibitory and stimulatory CM) were separated by precast 4-12% polyacrylamide gel electrophoresis (Genscript) under reducing conditions and stained with GelCode blue stain reagent (Thermo Scientific). Each gel lane was manually cut into 10 bands (Supplementary Fig.4). The bands were crushed into small fragments and submitted to in-gel trypsin digestion and peptide extraction. Peptide samples were analyzed on an Orbitrap Q Exactive mass spectrometer (ThermoFisher Scientific, Waltham, Massachusetts, USA) equipped with a DESI Omni Spray (Prosolia, Waters, Milford, Massachusetts, USA) used in nanospray mode and a nano-flow LC system (Easy-nLC II, ThermoFisher Scientific, Waltham, Massachusetts, USA). Peptides were separated on a Picofrit 25 cm x 0.75 µm i.d. column (New Objective, PF360-75-10- N-5, Woburn, Massachusetts, USA) packed in-house with C18 beads. Peptides were eluted with a gradient of 2-60% buffer B (100% ACN, 0.1% formic acid) at a flow rate of 300 nL/min.

Label-free proteomics was done in data-dependent acquisition (DDA), MS1 spectra were acquired from 400 to 2000 m/z at a resolution of 70000. The 20 most intense precursors were selected for fragmentation at 30 eV collision energy and the corresponding MS2 spectra were acquired at a resolution of 17500, using singly charged precursor ions, and ions of undefinable charged states were excluded from fragmentation. MaxQuant software (version 1.5.3.30) was used to analyze MS raw files. MS/MS spectra were searched against the human Uniprot FASTA database (Version 2016) and a database of common contaminants (247 entries) by the Andromeda search engine. Cysteine carbamidomethylation (IAA) and methionine oxidation were applied as variable modification.

Enzyme specificity was set to trypsin with a maximum of two missed cleavages and a minimum peptide length of 7 amino acids. A false discovery rate (FDR) of 1% was required for peptides and proteins. Peptides were identified was performed with an allowed initial precursor mass deviation of up to 7 ppm and an allowed fragment mass deviation of 20 ppm. Protein identification required at least one razor peptide. A minimum ratio of 1 was required for valid quantification events in MaxQuant’s Label-Free Quantification algorithm (MaxLFQ). Data were filtered for common contaminants and peptides only identified by side modification were excluded from further analysis. The mass spectrometry proteomics data have been deposited at the ProteomeXchange Consortium via the PRIDE partner repository with the dataset identifier PXD035770.

**Intra-pancreatic tumor injection**

Mice were anesthetized and left subcostal laparotomy performed. Spleen and pancreas were exposed, and the tumor cells suspension was injected in the pancreas. Organs were returned to the peritoneum (closed with 4-0 vicryl surgical suture), and the abdominal wall and skin sealed with a surgical staple. Tumor growth was evaluated by weekly abdominal palpation. Mice were promptly euthanized at the first signs of distress caused by tumour burden.

**Pharmacological treatments**

FC1199 cells (5x10^4^) were transplanted in the pancreas of six- to eight-week-old female C57BL/6 mice (Charles River Laboratories). Defactinib was administered orally, every day at the dose of 25 mg/kg twice/day or 30 mg/kg once/day. Erdafitinib was given orally every day at the dose of 12.5 mg/kg, twice/day, or 18 mg/kg, once/day. Control groups received the same volume of vehicle. Mice were weighed every other day as a measure of drug toxicity. Animals were euthanized 22 days after tumor injection, pancreas was weighed and collected for further analysis.

**Acute pancreatitis induction**

Acute pancreatitis was induced in 10-week-old C57BL/6 mice (Envigo, Correzzana, Italy). Briefly, mice were injected intraperitoneally with 100 µg/kg of caerulein (AnaSpec, Fremont, CA, USA) 6 times over 5 consecutive hours. The severity of acute injury was initially verified by measuring plasma pancreatic alpha-amylase and lipase using reflotron tests (Roche, Mannheim, Germany). After 24 hours ctrl and caerulein-treated mice were injected with 5x10^4^ MIAPaCa2 cells . Mice were euthanized 70 days after tumor cell injection.
